# A UK-wide analysis of the use of reversal agents in 198 patients on direct oral anticoagulants prior to urgent procedures

**DOI:** 10.1016/j.rpth.2026.106632

**Published:** 2026-05-08

**Authors:** HaemSTAR Collaborators, Molly Murphy, Michala Pettitt, Jasmine Makker, David J. Sutton, Bryar Kaddir, Andrew Doyle, Gillian Lowe, Rita Perry, Valerie Abbey, Alameldin Abdallah, Mohanad Abdelrahim, Ahmed Aboelmaaty, Ritika Abrol, Mutiu Adeyemo, Yasmin Ahmadi, Reem Ahmed, Maheen Ahsan, Laura Aiken, Lakhan Ajmeria, Asfana Akhi, Arun Alfred, Yasir Alhamdi, Adam Ali, Aamir Ali, Ariba Ali, Sahla Ali, Hazem Alnatour, Julia Anderson, Claire Anderson, Syed Abid Anjum, Laura Anthony, Saira Anwar, Muzna Aquil, Spatika Ashwini, Luke Attwell, Tin Aung, Thatoe Aung, Nazish Aurangzeb, Peter Baker, Sneha Balakrishnan, Mahina Baloch, Anjuli Banerjee, Gaynor Barrett, Giorgio Bartalucci, John Bartoli-Abdou, Ankhi Barua, Alexander Bashford, Karthik Basker, Gilda Bass, Jireh Ann Batac, Laura Batey, Chithrangani Batugedara, Ala Bawazir, Sue Beach, Lauren Beattie, Holly Beckett, Edward Belsham, Edward Benn, Gary Benson, Caroline Betts, Gopika Bhaskar, Sonal Bhatte, Rachael Biggart, Nuno Borges, Aparna Bose, Sara Boyce, Kubra Boza, Olivia Brooke, Stanley Broughton, Clare Brown, Emily Buchanan, Michael Buckton, Tom Bull, Carol Buttriss, Lorna Cain, Sedeshka Cakmak, Ipek Cakmak, Ross Campbell, Samantha Carrington, Matthew Carter, Daniel Castle, Dominique Chan-Lam, Shikha Chattree, Jia Ning Chen, Sue Lyn Chia, Shyamala Chinnabhandara, Catherine Chinnery, Tatiana Christmas, Deborah Clark, Charlotte Claydon, Ayodeji Coker, Freya Collings, Amy Cooper, Zignat Courtoux, Joanne Craig, Filippo Croce, Francesca Crolla, Henry Crosland, Kirsty Crozier, Ruth Cumber, Nicola Curry, Annie Curtis, Amapreet Davi, Olga Delacruz, Alison Delaney, Nicholas Denny, Ian Devanny, Prathiksha Dhanaraj, Saniya Dhawan, Jake Diack, Tsvetina Dimitrova, Suzanne Docherty, Maribeth Donaldson, Francesca Doughty, Samantha Drummond, Christie Drury, Madeleine Durkan, Portia Eardley, Timothy Ebsworth, Dewi Eden, Hugh Edwards, Katherine Elgar, Mohamed Elhadi, Lauren Ellis, Shereef Elmoamly, Astrid Etherington, Sally Evans, Edward Evans, Sophie Evans, Angharad Everden, Thomas Fail, Dina Fathoala, Joanna Fawcett, Cliona Flanaghan, Robbie Forsythe, Benjamin Franci, Rhian Fuge, Agata Gaertner, Sana Galfour, Julian Gertner, Milad Ghanikolahloo, Hannah Giles, Girgis Girgis, Poppy Godding, Shreya Goel, Tennelle Goulbourne, Benjamin Gray, Shane Grealish, Victoria Greer, Sharran Grey, Jessica Griffin, Sudarshan Gurung, La’ali Gutierrez, Cecilia Gyansah, Thura Htut, Anthony Hackett, Thwe Han, Elizabeth Hardiman, Laya Hariharan, Noor Haris, Emma Harris, Michelle Harrison, Alistair Hart, Nayeem Hasan, Aidan Haslam, Eman Hassan, Joanna Haughton, Myo Hein, Joannes Hermans, Charlotte Hickman, Joshua Hinds, Kelly Holton, Luke Hone, David Hopkins, Joanne Hoyle, Myint Htwe, Nathan Hutchinson-Jones, Kaustubh Ieetkar, Jack Illingworth, Mishal Iqbal, Hussein Jabbar, Nkemdirim Jacob, Laura James, Fatima Jamil, Nadeera Jayasekara, Sheila Jen, Scott Jenkins, Michael Joffe, Erith Jones, Elizabeth Jones, Benedict Jong, Emma Jongman, Sungjoo Kang, Angela Kanny, Mamatha Karanth, Farheen Karim, Richard Karim, Sajida Kazi, Ron Kerr, Rimsha Khan, Mohammed Khan, Jahanzeb Khawaja, Milo Kmonicek, Amy Knott, Laura Knox, Trushka Kolke, Neena Krishnan, Ayodeji Kunlere, Arka Kyaw, Grace Langford, Alex Langridge, Shafaq Lashari, Declan Leahy, Heather Leary, Claire Lentaigne, Francesca Leonforte, Sarah Lewis, Anand Lokare, Karyn Longmuir, Cheryl Ludlam, Apurva Lunia, Pyae Lwin, Ibrahim Maab, Ben MacLeod, Rhona Maclean, Catriona Mactier, Gihan Mahmoud, Tara Maisel, Herng Mak, Aritri Mandal, Stuart Mann, Zoha Mansoor, Claire Mapplebeck, Elizabeth Marrinan, Brigitta Marson, Natalie Martin, Navta Masand, Jon Massie, Abhinav Mathur, Bernard D. Maybury, Thomas Mayo, Michael McDonald, Ella McDuffus, Graham McIlroy, Anke Meess, Michelle Melly, Kainat Memon, Jerssy Mhike, Loredana Gabriela Mihailescu, Jayna Mistry, Amany Mohamed, Sarah Mohamed, Elnour Mohamed, Khatib Mohammad, Thet Mon, Shivir Y. Moosai, Samuel Morfett, James Morgan, Kerry Morrison, Kat Moss, Omar Mukhlif, Saman Mukhtar, Christopher Mullen, Laura Munglani, Jack Murrell, Komal Naeem, Taran Nandra, Stephanie Narine, Georgina Naylor, Lorna Neill, Loretta Ngu, Faiza Noor, Bright Nwatamole, Oluwasijibomi Olusola, Fatima Omrani, Nwe Oo, Zin Oo, Min Zin Oo, Dina Osman, Doha Osman, Hajer Oun, Oluwatamilore Oyewunmi, Andrew Page, Charles Page, Krushika Paleja, Athanasia Sinti Papadaki, Katrina Parsons, Fahan Patel, Aran Patel, Satya Lohitha Pedapati, Xavier Peer, Sam Pell, Kumari Perera, Joshua Peterson, James Phillips, Miriam Botelho Duarte Portela McKean, Matthew Powell, Mary Preston, Clare Proudfoot, Indu Punchihewa, Jackie Quinn, Maya Raj, Florije Raka, Chavin Ranasinghe, Benjamin Recaldin, Udaya Reddy, Wynona Reed, Hannah Reeves, James Riley, Ali Rismani, Jane Ritchie, Kristine Rivera, Lara Roberts, Patrick Roberts, Emily Robinson, Lisa Robinson, Philip Robson, Ryan Rodgers, Daniel W. Rogalsky, Victoria Rowson, Jackie Ruell, Jane Rutherford, Walaa Saad, Kamran Sabir, Malahat Saeed, Zahbia Saleem, Surenthini Salmon, Fady Samy, Rebecca Sarsam, Hayley Scott, Tom Scrivin, Moncef Seddiki, Sidhant Seth, Jainam Shah, Fatima Shakkak, Rebecca Shaw, Sam Sherratt-Mayhew, Amrit Shetty, Anjali Shrestha, Julia Sillito, David Simcox, Thomas Skinner, Sophie Smith, Shalini Solanki, David Sparksman, Vimal Stanley, Elizabeth Steven, Phillippa Stimpson, Maryam Subhan, Kajani Subhaskaran, Nadia Taha, Fatimah Talha, Balsam Tameemi, Juan Tan, Myuri Thanabalasingam, Alekh Thapa, Angela Theodoulou, Sarah Thislie, Kirstie Thorneycroft, Molly Thorpe, Ellen Tiffin, Sophie Todd, Nuthan Tom, Melissa Truman, Nomathamsanqa Tshuma, Olga Tsiamita, Micky Tsui, Mevish Ul-haq, Nataliia Vilinska, Hrushikesh Vyas, David Waddell, Rachel Walker, Sai Lon Wann, Vicki Ware, Alexander Wathen, Lucy Watkins, George Watkinson, Carys Watson, Simon Watt, Amy Webster, Katie White, Richard Whitmill, Charlotte Wild, Chloe Wilkinson, Gwennan Williams, Emma Williams, Julia Wolf, Hui Wong, Eleanor Wong, Stella Woodward, Rachel Wright, Philip Wright, Jane Wylie, Rehab Yassin, Dogan Yildiz, Idrees Zafar, Than Zaw, Mairi Walker, Laura Magill, Raza Alikhan, Phillip LR. Nicolson, Richard J. Buka

**Keywords:** anticoagulants, factor Xa inhibitors, surgical procedures, operative, radiology, interventional, prothrombin complex concentrates

## Abstract

**Background:**

Direct oral anticoagulants (DOACs) are widely prescribed but present challenges when patients require urgent invasive procedures. While idarucizumab is licensed for preprocedural reversal of dabigatran, 4-factor prothrombin complex concentrates (4F-PCCs) are often used off-label for oral factor (F)Xa inhibitors.

**Objectives:**

We aimed to describe real-world UK use of reversal agents prior to urgent procedures.

**Methods:**

We conducted a UK-wide audit of adults receiving andexanet alfa, idarucizumab, or 4F-PCC to reverse apixaban, dabigatran, edoxaban, or rivaroxaban, before urgent procedures between October 2020 and June 2023. Data were collected by members of HaemSTAR, a UK-wide network led by hematology resident doctors. Primary outcomes were (1) receipt of reversal ≤24 hours before procedure and (2) adherence to recommended dosing. Secondary outcomes included procedural bleeding risk, 90-day mortality, and 30-day thrombosis rate.

**Results:**

Data were collected on 198 patients, with 48% undergoing high-risk procedures. Median time from reversal to procedure was 1.6 hours and 76% took place within 24 hours. 4F-PCC was used in 87%, idarucizumab in 8%, and andexanet alfa in 5%. Tranexamic acid was administered in 20%, 90-day mortality was 24%, and thromboembolic events occurred in 3%, all following 4F-PCC.

**Conclusion:**

Reversal agents are frequently used prior to urgent procedures in direct oral anticoagulant–treated patients, including for low-risk interventions. There is limited evidence to support the off-label use of 4F-PCC; the risks, benefits, and cost effectiveness are uncertain. Delays to intervention, low use of tranexamic acid, and infrequent drug-level testing highlight opportunities to improve clinical workflows and inform future trials.

## Essentials

• Optimal management of patients on DOACs for urgent procedures is unclear.
• This UK-wide audit evaluated clinical practices for DOAC reversal prior to emergency procedures.
• Reversal agents are frequently administered even for procedures with low risk of bleeding.
• Standardization based on prospective evidence evaluating reversal agent use is needed.

## 1 Introduction

Direct oral anticoagulants (DOACs) are widely used, life-saving medications but are associated with a small, yet clinically important risk of major bleeding [1,2]. When patients taking DOACs require invasive procedures or surgery (excluding minimal-risk procedures that do not require interruption), temporary cessation of anticoagulation for between 24 and 96 hours is recommended, depending on the specific agent and renal function [3,4]. In patients requiring emergency or urgent procedures however, such interruption is often not possible. Patients anticoagulated with DOACs who undergo urgent invasive procedures are therefore at higher risk of bleeding than those undergoing planned surgery [5].

Given this increased bleeding risk, there is an intuitive rationale for attempting to reverse anticoagulation prior to urgent procedures, although whether this approach confers clinical benefit is uncertain. A meta-analysis of phase III atrial fibrillation trials examining mostly elective procedures in which anticoagulation was continued demonstrated lower rates of major bleeding with DOACs compared with warfarin, with the absolute risk decreasing from 3.3% to 2.0% [6]. However, high-quality data describing outcomes in patients undergoing urgent procedures, particularly with or without reversal agent use, are sparse.

To date, the only trial of a reversal agent for a DOAC that has included nonbleeding patients requiring urgent procedures is the Reversal Effects of Idarucizumab on Active Dabigatran (REVERSE-AD) study of idarucizumab for reversal of dabigatran [7]. This trial included 197 patients requiring urgent intervention. Normal hemostasis was observed in 93% but the trial lacked a comparator arm. On the basis of these data, idarucizumab was licensed for the reversal of dabigatran prior to urgent procedures [[7], [8], [9]]. In contrast, for patients taking apixaban, edoxaban, or rivaroxaban, no prospective trials have been completed in this setting. Andexanet alfa was licensed only for the treatment of major bleeding [10], while 4-factor prothrombin complex concentrate (4F-PCC), although widely used [11,12], is used off-label. However, in December 2025, due to a high risk of thromboembolic events, andexanet alfa was removed from manufacture and sale in the United States [13] but at the time of writing remains available in the rest of the world. Consequently, British guidelines recommend an expectant approach for patients taking FXa inhibitors, with reversal agents reserved for unexpected bleeding that occurs during the procedure [3]. Nonetheless, clinical concern regarding bleeding risk may prompt clinicians to intervene preemptively and this is an approach that is endorsed as an option in international guidelines [14].

We recently reported findings from a UK-wide audit of 2265 patients who received reversal agents for the management of DOAC-associated bleeding [12]. The primary aim of the project was to assess the appropriateness of reversal agent use. Although most patients met criteria for major bleeding, reversal was frequently administered long after symptom onset, hospital admission, and the last DOAC dose. That data collection also captured patients who received reversal agents prior to urgent procedures, and in the present article, we report those findings.

## 2 Methods

### 2.1 Patients and data collection

Data were collected during two 8-week windows (June to August 2022 and October to December 2023). Adults who received andexanet alfa, 4F-PCC (Beriplex or Octaplex), or idarucizumab, for reversal of the anticoagulant effect of either apixaban, dabigatran, edoxaban, or rivaroxaban prior to an urgent procedure between October 1, 2020, and June 30, 2023, were eligible for inclusion. The reason for reversal (urgent procedure or bleeding) was determined by the data contributor based on the clinical record.

Data were anonymized and entered into a secure online database using REDCap, managed by the University of Birmingham Centre for Prospective and Observational Studies (BiCOPS). Data entry was performed by members of HaemSTAR, a UK-wide network of hematology resident doctors interested in medical haematology. Procedures were categorized as minimal, low-moderate, or high risk for bleeding according to guidelines from the International Society on Thrombosis and Haemostasis (ISTH) [15]. Where patients underwent >1 procedure at the same time, the bleeding risk was classified according to the highest risk procedure.

### 2.2 Objectives

There were 2 primary outcomes. The first was the proportion of patients treated with a reversal agent who received treatment in accordance with the dosing schedule specified in the relevant summary of product characteristics. The second was the proportion of patients who received a preoperative reversal agent whose procedure was carried out within 24 hours. Secondary, exploratory outcomes included death within 90 days, thromboembolic events within 30 days, the proportion whose procedure was performed within 3 hours of reversal, red cell transfusion within 48 hours of reversal, and reduction in hemoglobin at 24 to 48 hours postreversal.

### 2.3 Statistical methods

Categorical variables were summarized using counts and percentages. Continuous variables were described using the mean and SD for normally distributed data or the median and IQR for nonnormally distributed data. Comparisons between 2 groups were performed using the *t*-test for normally distributed variables and the Mann–Whitney U-test for nonnormally distributed variables. Comparisons involving >2 groups with nonnormal distributions were performed using the Kruskal–Wallis test followed by Dunn post hoc comparisons. Mortality was visualized using Kaplan–Meier curves with statistical testing of difference by log-rank test. Hazard ratios for mortality were estimated using Cox proportional hazards regression. Odds ratios for binary outcomes were estimated using logistic regression. For both odds ratios and hazard ratios, corresponding 95% CIs were calculated. Missing data were assumed to be missing at random, and no imputation was performed. While pairwise comparisons were performed between all groups shown in figures, only statistical differences *P* < .05 are marked.

### 2.4 Ethics and governance

The protocol and data management plans were reviewed by the Birmingham Clinical Trials Unit and by the Caldicott Guardian at University Hospitals Birmingham NHS Foundation Trust. Each participating site reviewed and approved the project locally. All reviewers were satisfied that the project constituted an audit and did not require formal NHS research ethics approval.

## 3 Results

**Figure 1:**
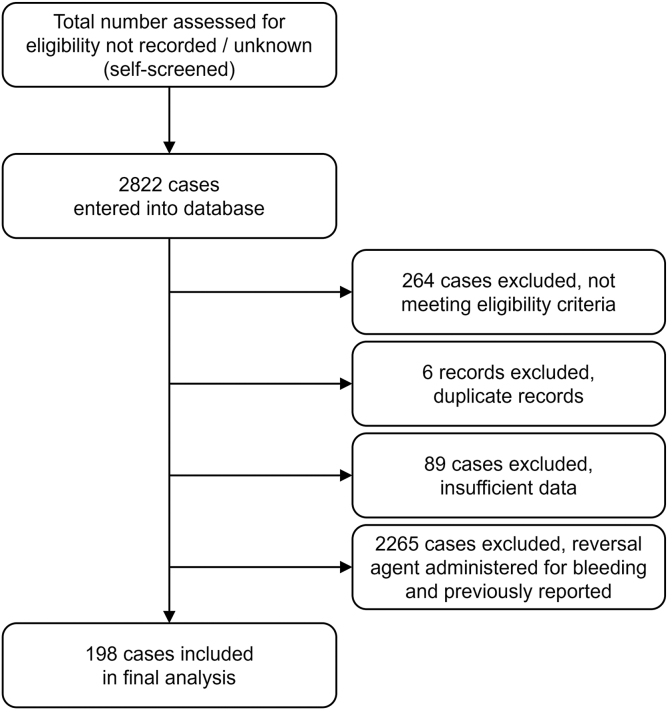
Study flowchart. Cases where reversal agents were used in the management of bleeding have been previously reported [12].

We gathered data on 198 patients from 38 UK hospitals who received either andexanet alfa or 4F-PCC (Beriplex or Octaplex) or idarucizumab, prior to an urgent procedure. The study flowchart is shown in Figure 1. In total, there were 202 procedures as 4 (2.0%) patients underwent 2 procedures at the same time point. Baseline patient characteristics including demographics, medical history, and laboratory findings, stratified by procedural bleeding risk are presented in Table 1.

**Table 1:** Demographics and baseline characteristics of patients receiving reversal agents prior to an urgent procedure stratified by procedural bleeding risk.

Procedural bleeding risk	High	Missing data	Low-moderate	Missing data	Minimal	Missing data	Unknown	Missing data	All patients
No. of patients	95 (48.0)		63 (31.8)		35 (17.7)		5 (2.5)		198
Age (y)^a^	75 (65.5-83.0)	0	78 (74.0-83.5)		78 (66-80.5)		79 (76-82)		78 (68.2-83.0)
Male sex	58 (61.1)	0	38 (60.3)		22 (62.9)		2 (40.0)		120 (60.6)
Ethnicity		22 (23.2)		12 (19.0)		9 (25.7)		0	
Asian	3 (3.2)		1 (1.6)		1 (2.9)		0		5 (2.5)
Black	1 (1.1)		1 (1.6)		1 (2.9)		0		3 (1.5)
Other	3 (3.2)		0		0		0		3 (1.5)
White	76 (80.0)		49 (77.8)		24 (38.6)		5		154 (77.8)
Comorbidities		0		0		0		0	
Active cancer	18 (18.9)		9 (14.3)		8 (22.9)		0		35 (17.7)
Arterial thrombosis	17 (17.9)		16 (25.4)		9 (25.7)		1 (20.0)		43 (21.7)
Atrial fibrillation	70 (73.7)		52 (82.5)		24 (68.6)		5 (100)		151 (76.3)
Dementia	6 (6.3)		5 (7.9)		0 (0.0)		0		11 (5.6)
Diabetes	25 (26.3)		14 (22.2)		7 (20.0)		1 (20.0)		47 (23.7)
Ischemic heart disease	23 (24.2)		16 (25.4)		13 (37.1)		0		57 (28.8)
Liver disease	3 (3.2)		1 (1.6)		2 (5.7)		0		6 (3.0)
Prior major bleed	3 (3.2)		8 (12.7)		1 (2.9)		0		12 (6.1)
Venous thrombosis	22 (23.2)		14 (22.2)		11 (31.4)		1 (20.0)		48 (24.2)
DOAC		0		0		0		0	
Apixaban	53 (55.8)		36 (57.1)		18 (51.4)		0		107 (54.0)
Dabigatran	6 (6.3)		3 (4.8)		3 (8.6)		5 (100)		17 (8.6)
Edoxaban	15 (15.8)		6 (9.5)		5 (14.2)		0		26 (13.1)
Rivaroxaban	21 (22.1)		18 (28.5)		9 (25.7)		0		48 (24.2)
Reduced dose DOAC^a^	25 (26.3)	0	18 (28.6)	1 (1.6)	12 (34.3)	0	2 (60.0)	0	57 (28.8)
Concurrent antiplatelet	8 (8.4)	0	5 (7.9)	0	1 (2.9)	0	0		17 (7.1)
Weight (kg)	74.0 (63.0-94.2)	23 (24.2)	76.5 (67.2-88.8)	19 (30.2)	76.5 (67.2-86.8)	9 (25.7)	56.5 (52.8-66.5)	1 (20.0)	76.0 (63.5-89.2)
DOAC level (ng/mL)	97 (67-202)	85 (89.5)	94 (61-127)	57 (90.5)	136 (89-186)	29 (82.9)	55	4 (80.0)	108 (61.5-192)
Reversal agent		0		0		0		0	
Andexanet alfa	3 (3.2)		6 (9.5)		1 (2.9)		0		10 (5.1)
400 mg bolus (low dose)	2 (66.7)		6 (100)		1 (100)				9 (45.5)
Idarucizumab	5 (5.3)		3 (4.8)		3 (8.6)		5 (100)		16 (8.1)
4F-PCC	87 (91.6)		6 (9.5)		1 (2.9)		0		172 (86.9)
Dose (IU)	2500 (2000-3000)	2 (2.3)	2000 (1500-2500)	1 (1.9)	2000 (1762-2500)	1 (3.2)			2010 (1725-3000)
Dose per kilogram (IU)	31.2 (25.5-43.4)	20 (23.0)	26.8 (21.2-33.1)	19 (35.1)	25.7 (23.9-35.2)	8 (25.8)			29.1 (24.6-41.7)
Presenting features									
Lowest systolic BP prior to reversal (mm Hg)	108 (93-130)	8 (8.4)	118 (103-137)	7 (11.1)	115 (96-127)	1 (2.9)	133.5 (105.2-162.5)	1 (20.0)	112 (97-131)
Highest heart rate prior to reversal (bpm)	88 (78-105)	8 (8.4)	91 (78-105)	7 (11.1)	88 (76-107)	1 (2.9)	109 (103.5-112.5)	1 (20.0)	90 (78-106)
Hemoglobin prior to reversal (g/L)	115 (95-112)	1 (1.1)	128 (112-144)	1 (1.1)	132 (106-148)	0	141 (125-144)	0	121 (103-142)
Creatinine at presentation (μmol/L)	99 (71-139)	0	92 (75-125)	1 (1.6)	112 (75-148)	0	71 (70-85)	0	99 (73-135)
Time from last dose of DOAC to reversal (h)	26.9 (15.9-32.0)	74 (77.9)	22.3 (10.6-24.0)	46 (73.0)	10.9 (7.1-25.3)	29 (82.3)	45.8 (45.8-45.8)	4 (80.0)	22.7 (12.3-29.4)
Time from reversal to procedure (h)	1.5 (0.5-4.4)	61 (64.2)	1.9 (1.5-4.1)	39 (61.9)	1.7 (0.7-2.7)	23 (65.7)		5 (100)	1.6 (0.8-4.1)

Percentages are calculated as % of patients for whom data were nonmissing. Categorical data presented as *n* (%) and continuous data as median (IQR).

BP, blood pressure; DOAC, direct oral anticoagulant; 4F-PCC, 4-factor prothrombin complex concentrate; IU, international units.

a Reduced-dose DOAC refers to any patient not on highest licensed dose of DOAC.

### 3.1 Procedural bleeding risk

95 (48.0%) patients underwent procedures classified as high bleeding risk, 63 (31.8%) as low-moderate risk, and 35 (17.7%) as minimal risk. Of 176 (88.9%) procedures where data were available, 160 (81.0%) were classified as unplanned. The most common indications for a procedure or surgery were hernia repair (32, 16.2%), bowel resection (29, 14.6%), and chest drain insertion (21, 10.4%) (Supplementary Table 1). A full list of the details of each patient, the procedure, and indication is shown in Supplementary Table 2.

### 3.2 Timing of reversal agent administration and procedure

**Figure 2:**
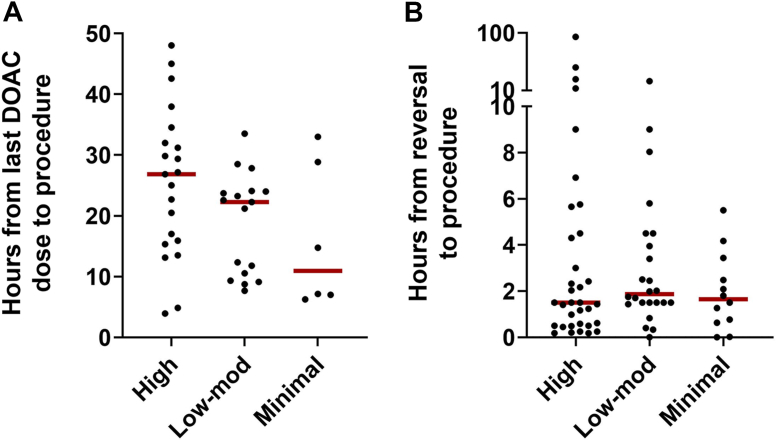
Timing of reversal agent administration according to procedural bleeding risk. (A) Time from last reported dose of anticoagulant to reversal agent administration (*n* = 45). (B) Time from reversal agent administration to procedure (*n* = 70). Low-mod, low-moderate. Statistical testing of difference by Kruskal–Wallis test with Dunn multiple comparisons. DOAC, direct oral anticoagulant.

Of 45 (22.7%) patients where data were available, the median time from last dose of DOAC to reversal agent administration was 22.7 hours (IQR, 12.3-29.4 hours) (Figure 2A). Of 70 (35.4%) patients where data were available, 53 (75.7%) underwent a procedure within 24 hours of reversal and 39 (55.7%) within 3 hours (Figure 2B). The median time from reversal agent administration to procedure was 1.6 hours (IQR, 0.8-4.1 hours) (Figure 2B). There were no significant differences in these timings according to procedural bleeding risk (Figure 2). Of 72 (36.4%) patients with time of procedure recorded, 36 (50.0%) took place between 6:00 pm and 6:00 am and 16 (22.2%) took place between 12:00 am and 6:00 am.

### 3.3 Reversal agents

The most frequently used reversal agent was 4F-PCC (172, 86.9%), followed by idarucizumab (16, 8.1%) and andexanet alfa (10, 5.1%). Andexanet alfa was administered to 10 patients, 5 (50%) of whom were anticoagulated with apixaban, 1 (10%) edoxaban, and 4 (40%) rivaroxaban. One (10%) patient received a high-dose bolus of 800 mg, followed by a high-dose infusion of 960 mg. Eight (80%) patients received a low-dose bolus of 400 mg, followed by a low-dose infusion of 480 mg, while 1 (10%) patient who received a low-dose bolus did not receive an infusion.

Of the 16 patients who received idarucizumab, all had a history of treatment with dabigatran; 15 (93.8%) of these patients received the licensed 5 g dose, and 1 (6.2%) received 2.5 g. No patients received a second dose of idarucizumab.

**Figure 3:**
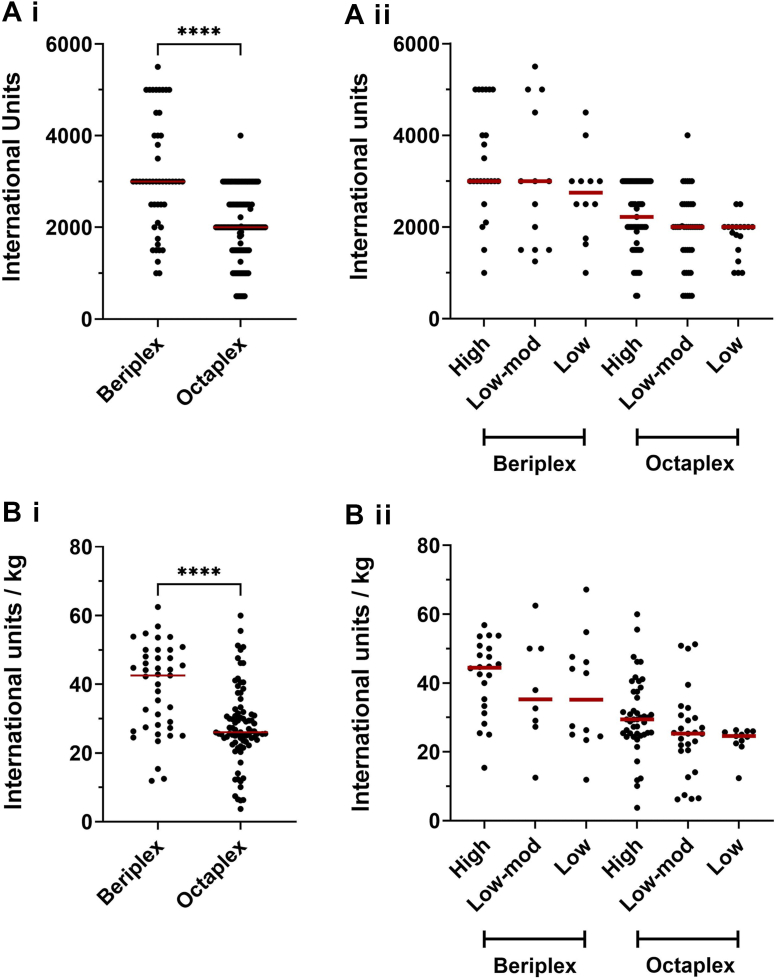
Dosing of 4-factor prothrombin complex concentrate (4F-PCC). (Ai) Absolute dose of 4F-PCC stratified by formulation (Beriplex or Octaplex). (Aii) Absolute dose of 4F-PCC stratified by formulation and procedural bleeding risk (*n* = 168). (B) As for (A) but 4F-PCC dose presented as per kilogram (*n* = 125). Low-mod, low-moderate. Statistical testing of difference for (Ai) and (Bi) by Mann–Whitney U-test. Statistical testing of difference for (Aii) and (Bii) by Kruskal–Wallis test with Dunn multiple comparisons. ∗∗∗∗*P* < .0001.

Of the 172 patients who received 4F-PCC, 53 (30.8%) received Beriplex and 119 (69.2%) received Octaplex. One patient received a dose above the recommended 5000 IU maximum for Beriplex, and another received a dose above the 3000 IU maximum for Octaplex. The median dose of 4F-PCC was 2010 IU (IQR, 1725-3000 IU), and the median dose per kilogram was 29.1 IU/kg (IQR, 24.6-41.7 IU/kg) (Table 1). The overall median dose of Beriplex was significantly higher than that of Octaplex (3000 IU [IQR, 2500-4000 IU] vs 2000 [IQR, 1500-2500 IU]; *P* < .0001) as was the dose per kilogram (42.7 IU/kg [IQR, 27.5-49.5 IU] vs 26.0 [IQR, 23.4-31.4 IU]; *P* < .0001) (Figure 2Ai, Bi). The proportion of patients receiving higher dose 4F-PCC (as measured by both dose per kilogram) and absolute dose (defined for both as above vs below or equal to the median) was significantly higher for high-risk procedures (Supplementary Tables 3 and 4). Although the same pattern was observed when Beriplex and Octaplex were analyzed separately, and as continuous data, this was not statistically significant (Figure 3); 40 (20.2%) patients received tranexamic acid (high risk: 23/95 [24.2%], low-moderate: 9/63 [14.3%], minimal: 8/35 [12.7%]). 14 (7.1%) patients were prescribed antiplatelet medication prior to the procedure, and none received a platelet transfusion prior to the procedure.

### 3.4 Clinical advice

Of 176 patients for whom data on clinical advice were available, advice from a hematologist was sought in 130 (73.9%). Of these, 11 (8.5%) were also discussed with other specialties, including gastroenterology, general surgery, intensive care, respiratory, gynecology, interventional radiology, cardiology, and urology.

### 3.5 Drug levels

DOAC levels were reported in 23 (13.1%) patients. The median level was 108 ng/mL (IQR, 62-192 ng/mL) and 3 of 23 (13.0%) patients had a level of <50 ng/mL. We did not collect data on whether this level was used to guide management.

### 3.6 Outcomes

**Figure 4:**
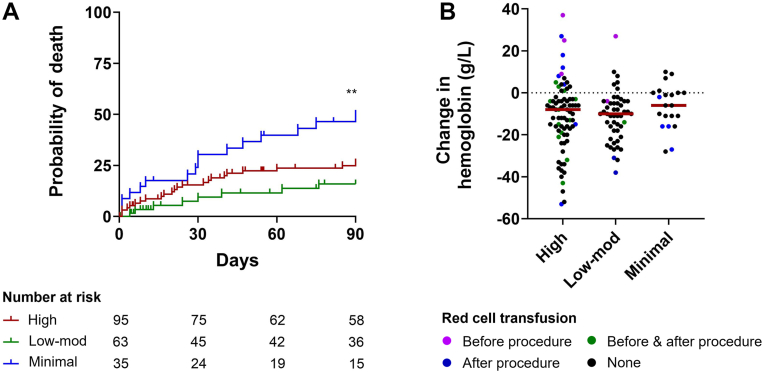
Outcomes after reversal agent administration. (A) Survival after reversal agent administration stratified by procedural bleeding risk. Statistical testing of difference between curves by log-rank test. (B) Change in hemoglobin (g/L) from preprocedural level to postprocedure. Colors indicate patients that were transfused either before (magenta), after (blue), or before and after (green) the procedure (*n* = 151). Statistical testing of difference by 1-way anova. ∗∗*P* < .01. Low-mod, low-moderate.

Clinical outcomes according to procedural bleeding risk are summarized in Table 2. At 90 days postreversal, 48 (24.2%) patients had died. Mortality was highest after minimal bleeding risk procedures (Table 2 and Figure 4A). Of those who had minimal bleeding risk procedures and died, procedures were frequently performed in the context of significant comorbidities such as active cancer (6, 37.5%), management of renal failure (line insertions: 4, 25.0%), and heart failure (biventricular assist device insertion: 1, 6.3%) Additionally, the median creatinine in this group was 125 μmol/L (IQR, 77-164 μmol/L) and 3 (18.8%) patients had a creatinine of >300 μmol/L.

**Table 2:** Clinical outcomes.

Procedural bleeding risk	High	Low-moderate	Minimal	Unknown	All patients	Effect estimate, HR/OR (95% CI)^a^
No. of patients	95 (48.0)	63 (31.8)	35 (17.7)	5 (2.5)	198	
Death within 90 d	23 (24.2)	8 (12.7)	16 (45.7)	1 (20.0)	48 (24.2)	High vs min: HR, 0.46 (0.24-0.88) High vs low-mod: HR, 1.81 (0.81-4.04) Low-mod vs min: HR, 0.26 (0.11-0.60)
Thromboembolic event within 30 d	5 (5.3)	0	1 (2.9)		6 (3.0)	Not estimated (few events)
Left atrial thrombus	1 (1.1)					
Myocardial infarction	1 (1.1)					
Pulmonary embolism	3 (3.3)					
Stroke			1 (2.9)			
≥1 red cell units transfused within 48 h	20 (21.1)	2 (3.2)	5 (14.3)	0	27 (13.8)	High vs min: OR, 1.60 (0.29-8.45) High vs low-mod: OR, 8.13 (2.25-52.21) Low-mod vs min: OR, 0.20 (0.03-0.97)
≥2 red cell units transfused within 48 h	12 (12.6)	1 (1.6)	2 (5.7)	0	15 (75.8)	High vs min: OR, 2.39 (0.61-15.86) High vs low-mod: OR, 8.96 (1.70-165.46) Low-mod vs min: OR, 0.27 (0.01-2.88)
≥20 g/L fall in hemoglobin at 24-48 h	*n* = 80, 19 (23.8)	*n* = 50, 12 (19.0)	*n* = 21, 2 (5.7)	*n* = 5, 1 (20.0)	*n* = 156, 34 (21.8)	High vs min: OR, 4.13 (1.11-26.83) High vs low-mod: OR, 1.06 (0.48-2.43) Low-mod vs min: OR, 3.88 (0.98-25.98)
Composite of ≥2 red cell units transfused within 48 h OR ≥20 g/L fall in hemoglobin at 24-48 h	*n* = 80, 27 (28.4)	*n* = 50, 12 (19.0)	*n* = 21, 4 (11.4)	*n* = 5, 1 (20.0)	*n* = 156, 44 (22.2)	High vs min: OR, 3.08 (1.09-11.07) High vs low-mod: OR, 1.69 (0.79-3.75) Low-mod vs min: OR, 1.82 (0.58-6.97)

HR, hazards ratio; low-mod, low-moderate bleeding risk procedure; min, minimal bleeding risk procedure; OR, odds ratio.

a Effect estimates are HRs for mortality (Cox proportional hazards model) and ORs for other outcomes (logistic regression), with high procedural bleeding risk as the reference group.

Six (3.0%) patients experienced a thromboembolic event within 30 days of reversal. There were 3 arterial events: 1 stroke after 10 days (after pleural drain in a patient with active cancer), 1 myocardial infarction after 14 days (after large bowel resection), and 1 atrial thrombus after 15 days (after pelvic fracture repair). There were 3 venous events all of which were pulmonary embolisms: at 13 days (after femoral pseudoaneurysm exploration), 14 days (after knee replacement revision), and 25 days (after evacuation of an infected skin haematoma). All events occurred in patients who received 4F-PCC (Octaplex, 1 [pulmonary embolism]; Beriplex, 5). Therapeutic, oral anticoagulation was restarted in 156 (79%) patients at a median of 4 days (IQR, 2-8 days) following reversal. Four thromboembolic events (66.7%) occurred in patients who had restarted oral anticoagulation.

Patients undergoing high-risk bleeding procedures were more likely to receive ≥2 red cell units and/or have a fall in hemoglobin of ≥20 g/L in the 48 hours after the procedure (Table 2). Sixteen (6.6%) patients received a red cell transfusion prior to the procedure (high risk, 12/95 [12.6%]; low-moderate, 3/35 [8.6%]; and minimal, 1/35 [2.9%]). Of 5 patients, 4 (80%) patients who received a red cell transfusion after minimal bleeding risk procedures had a history of preprocedural bleeding. One underwent cystoscopy for hematuria secondary to known bladder cancer, 2 underwent chest drain insertion for hemothorax, and 1 underwent examination under anesthesia for rectal bleeding. The other patient had undergone biventricular assist device insertion and had had a preprocedural hemoglobin of 88 g/L.

## 4 Discussion

In this UK-wide audit of 198 DOAC-treated patients, we observed frequent preprocedural use of anticoagulant reversal agents, predominantly 4F-PCC. Although idarucizumab is the only licensed agent for reversal (of dabigatran) prior to urgent procedures, reversal agents were commonly administered to patients anticoagulated with oral FXa inhibitors prior to wide range of procedures, including many not classified as high bleeding risk. Over half of patients received reversal before procedures deemed low or low-moderate bleeding risk by ISTH guidance [15], including hernia repair, appendectomy, chest drain insertion, and pacemaker implantation. Notably, pacemaker insertion is a procedure for which continuation of anticoagulation has been shown to be noninferior or beneficial in randomized trials [16,17]. Although we lack a denominator to determine how frequently procedures are undertaken without reversal, these findings suggest that administration of reversal agents prior to procedures is common. However, aside from patients treated with idarucizumab, use of reversal agents prior to procedures is off-label and not consistent with UK guidelines [3]. It is therefore unclear why reversal agents are used, particularly prior to minimal or low-moderate bleeding risk procedures, and further research is required to understand decision making.

Evidence guiding the management of DOAC-treated patients requiring urgent procedures is limited. In a European registry study of 478 patients treated between 2013 and 2015, urgent procedures were frequently delayed; 67% were major bleeding risk procedures, yet only 16% received hemostatic agents, and excessive bleeding occurred in 13% [18]. These data suggest that although there is a substantial risk in a minority of cases, many procedures are safely performed without reversal. A more recent retrospective study across 7 hospitals in the Netherlands included 62 patients who received reversal agents for DOACs prior to procedures (39 idarucizumab, 20 4F-PCC, and 3 andexanet alfa). This study showed similarly varied indications and dosing of PCC as in our cohort [19]. In the Randomized Evaluation of Long-Term Anticoagulation Therapy (RE-LY) trial of dabigatran versus warfarin for the prevention of stroke in atrial fibrillation, 353 patients underwent urgent procedures, and despite the absence of a specific dabigatran reversal agent at the time, bleeding and thromboembolic outcomes did not differ between dabigatran- and warfarin-treated patients [5]. In the REVERSE-AD trial of idarucizumab for dabigatran reversal, 20% of patients undergoing urgent procedures required red cell transfusion within 2 days [7] comparable with the 21% observed in our high-risk cohort, most of whom received 4F-PCC. Differences in pharmacokinetics of dabigatran and oral FXa inhibitors, and timing of last anticoagulant dose limit direct comparison. However, in general, there is no strong evidence that administration of reversal agents improves patient outcomes and that the benefits outweigh the risks.

In this cohort, the median time from reversal to procedure was 1.6 hours and only just over half of patients underwent their procedure within 3 hours. First, this raises questions about the urgency of procedures in some cases. In our view, it would be preferable to give the reversal agent as close to the procedure as possible as this better guarantees that the procedure is actually going to take place. Second, given the short half-life of coagulation factors, particularly FVII in 4F-PCC, administration as close to the procedure as possible would be expected to maximize hemostatic efficacy.

Furthermore, the median time from last DOAC dose to reversal was 22.7 hours, suggesting that many patients may already have had low residual anticoagulant levels at the time of reversal. Despite this, DOAC drug levels were infrequently measured, consistent with our previous findings in bleeding patients [12]. Given the observed delays, measurement of drug levels would have been feasible in many cases and could support more targeted decision making. However, although levels are informative, the impact of using urgent drug levels to guide the timing of procedures is underresearched. At a population level, it is plausible that access to drug level testing could have unintended consequences such as delays in procedures and potentially undue harm. Further evaluation of the real-world impact is required.

Tranexamic acid was administered in only 20% of cases. Although patients receiving therapeutic anticoagulation have been underrepresented in randomized trials of tranexamic acid, it is well established to reduce surgical blood loss [20,21], without an associated increase in thromboembolic risk [22], and its broader use in this context should be encouraged through education and clinical systems [23].

In this cohort, thromboembolic events were reported in 6 patients, all occurring at least 10 days after the procedure. The incidence of hospital acquired venous thromboembolism of 1.5% is comparable with generally observed rates postsurgery [24,25]. There are limited data on the incidence of arterial thrombosis after emergency surgery but the incidence of stroke in patients with atrial fibrillation in the 30 days postelective surgery has been reported as 0.7% [26]. The rates of thromboembolism in this cohort seem substantially lower than the rates after reversal agent administration in patients who are bleeding [12,27]. However, these data should not be viewed as a demonstration of the safety of agents, particularly 4F-PCC, as this is a small retrospective cohort that is not powered to sensitively detect events.

There are several further limitations to this analysis. The retrospective design relies on chart review and is therefore susceptible to missing data, most commonly due to incomplete documentation. Our primary outcome of time to procedure was available for assessment in only a third of cases, likely due to incomplete documentation. Similarly, the time from last DOAC dose could be ascertained in just under a quarter of patients. However, these data are likely missing at random, and the available information is expected to reasonably reflect real-world practice. A further limitation of retrospective assessment of bleeding outcomes is the lack of high-fidelity, routinely collected measures of procedural and periprocedural blood loss; operator’s assessment of hemostasis, an important clinical outcome, was not captured in this dataset. Interpretation is also limited by the highly heterogeneous nature of the cohort, which included a wide range of procedures performed for diverse indications. Moreover, the indication for reversal agent administration, either prior to an urgent procedure or for bleeding, was assigned by contributors based on clinical records. As a result, some cases categorized as preprocedural reversal may in fact have involved treatment of active bleeding, such as pleural drain insertion for hemothorax. Finally, although we captured several factors likely to influence decision making, including bleeding risk, renal function, and time from last anticoagulant dose, real-world practice is heavily influenced by nuanced clinical judgment and multidisciplinary discussion. These elements are difficult to quantify, and no retrospective study can fully capture the complexity of decision making in such scenarios.

These findings highlight substantial variation in practice and suggest that reversal is often used in scenarios with limited supporting evidence. Upcoming and ongoing trials [28,29] will be crucial in determining whether reversal confers clinical benefit. However, these studies will be challenging due to the rarity of patients, heterogeneity of presentations, and low occurrence of clinically meaningful events. Pragmatic standard-of-care comparator arms may further dilute observable effects if off-label 4F-PCC use is already widespread.

In view of the uncertainty of benefit, possibility for harm, and cost of reversal agents, treatment decisions should be made with full knowledge of the shortcomings in evidence. However, decision making in medicine is imperfect, and we should acknowledge that there may be benefits to using reversal agents in the preprocedural setting that go beyond their hemostatic activity. This is rarely acknowledged or discussed but, for example, timely administration of a reversal agent may provide reassurance to an operator, prompting them to undertake an intervention earlier than would otherwise have been acceptable and leading to improved outcomes.

In conclusion, this national audit demonstrates frequent preprocedural use of reversal agents for DOACs, including for procedures with low or moderate bleeding risk. Although this is UK-specific data, given the paucity of prospective data to guide practice, our findings are probably consistent with practice in other health care systems. Given limited evidence and guideline support for many of these scenarios, more precise approaches incorporating timing, drug levels, and adjunctive therapies are needed. Prospective studies will be essential to define optimal management and to determine whether reversal improves meaningful clinical outcomes.

## Appendix 1

### HaemSTAR Collaborators

Molly Murphy, Michala Pettitt, Jasmine Makker, David J Sutton, Bryar Kaddir, Andrew Doyle, Gillian Lowe, Rita Perry, Valerie Abbey, Alameldin Abdallah, Mohanad Abdelrahim, Ahmed Aboelmaaty, Ritika Abrol, Mutiu Adeyemo, Yasmin Ahmadi, Reem Ahmed, Maheen Ahsan, Laura Aiken, Lakhan Ajmeria, Asfana Akhi, Arun Alfred, Yasir Alhamdi, Adam Ali, Aamir Ali, Ariba Ali, Sahla Ali, Hazem Alnatour, Julia Anderson, Claire Anderson, Syed Abid Anjum, Laura Anthony, Saira Anwar, Muzna Aquil, Spatika Ashwini, Luke Attwell, Tin Aung, Thatoe Aung, Nazish Aurangzeb, Peter Baker, Sneha Balakrishnan, Mahina Baloch, Anjuli Banerjee, Gaynor Barrett, Giorgio Bartalucci, John Bartoli-Abdou, Ankhi Barua, Alexander Bashford, Karthik Basker, Gilda Bass, Jireh Ann Batac, Laura Batey, Chithrangani Batugedara, Ala Bawazir, Sue Beach, Lauren Beattie, Holly Beckett, Edward Belsham, Edward Benn, Gary Benson, Caroline Betts, Gopika Bhaskar, Sonal Bhatte, Rachael Biggart, Nuno Borges, Aparna Bose, Sara Boyce, Kubra Boza, Olivia Brooke, Stanley Broughton, Clare Brown, Emily Buchanan, Michael Buckton, Tom Bull, Carol Buttriss, Lorna Cain, Sedeshka Cakmak, Ipek Cakmak, Ross Campbell, Samantha Carrington, Matthew Carter, Daniel Castle, Dominique Chan-Lam, Shikha Chattree, Jia Ning Chen, Sue Lyn Chia, Shyamala Chinnabhandara, Catherine Chinnery, Tatiana Christmas, Deborah Clark, Charlotte Claydon, Ayodeji Coker, Freya Collings, Amy Cooper, Zignat Courtoux, Joanne Craig, Filippo Croce, Francesca Crolla, Henry Crosland, Kirsty Crozier, Ruth Cumber, Nicola Curry, Annie Curtis, Amapreet Davi, Olga Delacruz, Alison Delaney, Nicholas Denny, Ian Devanny, Prathiksha Dhanaraj, Saniya Dhawan, Jake Diack, Tsvetina Dimitrova, Suzanne Docherty, Maribeth Donaldson, Francesca Doughty, Samantha Drummond, Christie Drury, Madeleine Durkan, Portia Eardley, Timothy Ebsworth, Dewi Eden, Hugh Edwards, Katherine Elgar, Mohamed Elhadi, Lauren Ellis, Shereef Elmoamly, Astrid Etherington, Sally Evans, Edward Evans, Sophie Evans, Angharad Everden, Thomas Fail, Dina Fathoala, Joanna Fawcett, Cliona Flanaghan, Robbie Forsythe, Benjamin Franci, Rhian Fuge, Agata Gaertner, Sana Galfour, Julian Gertner, Milad Ghanikolahloo, Hannah Giles, Girgis Girgis, Poppy Godding, Shreya Goel, Tennelle Goulbourne, Benjamin Gray, Shane Grealish, Victoria Greer, Sharran Grey, Jessica Griffin, Sudarshan Gurung, La’ali Gutierrez, Cecilia Gyansah, Thura Htut, Anthony Hackett, Thwe Han, Elizabeth Hardiman, Laya Hariharan, Noor Haris, Emma Harris, Michelle Harrison, Alistair Hart, Nayeem Hasan, Aidan Haslam, Eman Hassan, Joanna Haughton, Myo Hein, Joannes Hermans, Charlotte Hickman, Joshua Hinds, Kelly Holton, Luke Hone, David Hopkins, Joanne Hoyle, Myint Htwe, Nathan Hutchinson-Jones, Kaustubh Ieetkar, Jack Illingworth, Mishal Iqbal, Hussein Jabbar, Nkemdirim Jacob, Laura James, Fatima Jamil, Nadeera Jayasekara, Sheila Jen, Scott Jenkins, Michael Joffe, Erith Jones, Elizabeth Jones, Benedict Jong, Emma Jongman, Sungjoo Kang, Angela Kanny, Mamatha Karanth, Farheen Karim, Richard Karim, Sajida Kazi, Ron Kerr, Rimsha Khan, Mohammed Khan, Jahanzeb Khawaja, Milo Kmonicek, Amy Knott, Laura Knox, Trushka Kolke, Neena Krishnan, Ayodeji Kunlere, Arka Kyaw, Grace Langford, Alex Langridge, Shafaq Lashari, Declan Leahy, Heather Leary, Claire Lentaigne, Francesca Leonforte, Sarah Lewis, Anand Lokare, Karyn Longmuir, Cheryl Ludlam, Apurva Lunia, Pyae Lwin, Ibrahim Maab, Ben MacLeod, Rhona Maclean, Catriona Mactier, Gihan Mahmoud, Tara Maisel, Herng Mak, Aritri Mandal, Stuart Mann, Zoha Mansoor, Claire Mapplebeck, Elizabeth Marrinan, Brigitta Marson, Natalie Martin, Navta Masand, Jon Massie, Abhinav Mathur, Bernard Maybury, Thomas Mayo, Michael McDonald, Ella McDuffus, Graham McIlroy, Anke Meess, Michelle Melly, Kainat Memon, Jerssy Mhike, Loredana Gabriela Mihailescu, Jayna Mistry, Amany Mohamed, Sarah Mohamed, Elnour Mohamed, Khatib Mohammad, Thet Mon, Shivir Moosai, Samuel Morfett, James Morgan, Kerry Morrison, Kat Moss, Omar Mukhlif, Saman Mukhtar, Christopher Mullen, Laura Munglani, Jack Murrell, Komal Naeem, Taran Nandra, Stephanie Narine, Georgina Naylor, Lorna Neill, Loretta Ngu, Faiza Noor, Bright Nwatamole, Oluwasijibomi Olusola, Fatima Omrani, Nwe Oo, Zin Oo, Min Zin Oo, Dina Osman, Doha Osman, Hajer Oun, Oluwatamilore Oyewunmi, Andrew Page, Charles Page, Krushika Paleja, Athanasia Sinti Papadaki, Katrina Parsons, Fahan Patel, Aran Patel, Satya Lohitha Pedapati, Xavier Peer, Sam Pell, Kumari Perera, Joshua Peterson, James Phillips, Miriam Botelho Duarte Portela McKean, Matthew Powell, Mary Preston, Clare Proudfoot, Indu Punchihewa, Jackie Quinn, Maya Raj, Florije Raka, Chavin Ranasinghe, Benjamin Recaldin, Udaya Reddy, Wynona Reed, Hannah Reeves, James Riley, Ali Rismani, Jane Ritchie, Kristine Rivera, Lara Roberts, Patrick Roberts, Emily Robinson, Lisa Robinson, Philip Robson, Ryan Rodgers, Daniel W Rogalsky, Victoria Rowson, Jackie Ruell, Jane Rutherford, Walaa Saad, Kamran Sabir, Malahat Saeed, Zahbia Saleem, Surenthini Salmon, Fady Samy, Rebecca Sarsam, Hayley Scott, Tom Scrivin, Moncef Seddiki, Sidhant Seth, Jainam Shah, Fatima Shakkak, Rebecca Shaw, Sam Sherratt-Mayhew, Amrit Shetty, Anjali Shrestha, Julia Sillito, David Simcox, Thomas Skinner, Sophie Smith, Shalini Solanki, David Sparksman, Vimal Stanley, Elizabeth Steven, Phillippa Stimpson, Maryam Subhan, Kajani Subhaskaran, Nadia Taha, Fatimah Talha, Balsam Tameemi, Juan Tan, Myuri Thanabalasingam, Alekh Thapa, Angela Theodoulou, Sarah Thislie, Kirstie Thorneycroft, Molly Thorpe, Ellen Tiffin, Sophie Todd, Nuthan Tom, Melissa Truman, Nomathamsanqa Tshuma, Olga Tsiamita, Micky Tsui, Mevish Ul-haq, Nataliia Vilinska, Hrushikesh Vyas, David Waddell, Rachel Walker, Sai Lon Wann, Vicki Ware, Alexander Wathen, Lucy Watkins, George Watkinson, Carys Watson, Simon Watt, Amy Webster, Katie White, Richard Whitmill, Charlotte Wild, Chloe Wilkinson, Gwennan Williams, Emma Williams, Julia Wolf, Hui Wong, Eleanor Wong, Stella Woodward, Rachel Wright, Philip Wright, Jane Wylie, Rehab Yassin, Dogan Yildiz, Idrees Zafar, Than Zaw, Mairi Walker, Laura Magill, Raza Alikhan, Phillip LR Nicolson, Richard J Buka.
