# Supplementary material for: A UK-wide analysis of the use of reversal agents in 198 patients on direct oral anticoagulants prior to urgent procedures
Source: Res Pract Thromb Haemost. 2026 May 8;10(4):106632. doi: 10.1016/j.rpth.2026.106632 (PMC13264241; doi:10.1016/j.rpth.2026.106632)
Supplement: Supplementary Tables 1 to 4 [file mmc1.docx]

# Supplemental materials

**Supplementary Table 1. Procedures performed organised by anatomical location or specialty, specific procedure, and indication.** BiVAD: biventricular assist device; CABG: coronary artery bypass graft; CVC: central venous catheter; CT: computed tomography; DOAC: direct oral anticoagulant; F: female; ICD: intercostal drain; JAK2+ MPN: JAK2-positive myeloproliferative neoplasm; M: male; μmol/L: micromoles per liter; NSTEMI: non-ST-elevation myocardial infarction; VATS: video-assisted thoracoscopic surgery; VP shunt: ventriculoperitoneal shunt.

| **Anatomic location or specialty** | **Procedure** | **Indication** | **Total** | **% of total** | **Subtotal** | **% of total** | **Total** | **Andexanet alfa** | **Idarucizumab** | **4F-PCC** |
| --- | --- | --- | --- | --- | --- | --- | --- | --- | --- | --- |
| **Abdominal** |  |  | 91 | 46.0 |  |  |  | 7 | 3 | 81 |
|  | Appendicectomy |  |  |  | 9 | 4.5 |  |  |  |  |
|  |  | Appendicitis |  |  |  |  | 6 | 1 | 0 | 5 |
|  |  | Appendicitis - perforated |  |  |  |  | 3 | 1 | 0 | 2 |
|  | Bowel resection |  |  |  | 29 | 14.6 |  |  |  |  |
|  |  | Bowel ischemia |  |  |  |  | 4 | 0 | 0 | 4 |
|  |  | Bowel obstruction |  |  |  |  | 5 | 0 | 0 | 5 |
|  |  | Bowel perforation |  |  |  |  | 1 | 0 | 0 | 1 |
|  |  | Bowel perforation and ischemic leg |  |  |  |  | 1 | 0 | 0 | 1 |
|  |  | Bowel obstruction and appendicitis |  |  |  |  | 1 | 0 | 0 | 1 |
|  |  | Large bowel obstruction |  |  |  |  | 3 | 0 | 0 | 3 |
|  |  | Large bowel perforation |  |  |  |  | 2 | 0 | 0 | 2 |
|  |  | Large bowel perforation - impending |  |  |  |  | 1 | 0 | 0 | 1 |
|  |  | Rectal prolapse |  |  |  |  | 1 | 0 | 0 | 1 |
|  |  | Sigmoid volvulus |  |  |  |  | 1 | 0 | 0 | 1 |
|  |  | Small bowel obstruction |  |  |  |  | 5 | 1 | 0 | 4 |
|  |  | Strangulated femoral hernia |  |  |  |  | 1 | 0 | 0 | 1 |
|  |  | Unknown |  |  |  |  | 2 | 0 | 0 | 2 |
|  |  | Ventral hernia |  |  |  |  | 1 | 0 | 0 | 1 |
|  | Bowel resection + lower limb embolectomy |  |  |  | 1 | 0.5 |  |  |  |  |
|  |  | Bowel perforation and ischemic leg |  |  |  |  | 1 | 0 | 0 | 1 |
|  | Cholecystectomy |  |  |  | 2 | 1.0 |  |  |  |  |
|  |  | Cholecystitis |  |  |  |  | 2 | 1 | 0 | 1 |
|  | Drain |  |  |  |  |  |  |  |  |  |
|  |  | Ascites |  |  | 6 | 3.0 | 6 | 0 | 0 | 3 |
|  |  | Intrabdominal abscess |  |  | 1 | 0.5 | 1 | 0 | 0 | 1 |
|  |  | Liver abscess |  |  | 2 | 1.0 | 2 | 0 | 0 | 2 |
|  | Hernia repair |  | 31 | 15.7 |  |  |  |  |  |  |
|  |  | Hernia |  |  |  |  | 1 | 0 | 0 | 1 |
|  |  | Incarcerated hernia |  |  |  |  | 12 | 3 | 2 | 7 |
|  |  | Incarcerated hernia and small bowel obstruction |  |  |  |  | 1 | 0 | 0 | 1 |
|  |  | Small bowel obstruction |  |  |  |  | 5 | 0 | 1 | 4 |
|  |  | Strangulated hernia |  |  |  |  | 9 | 0 | 0 | 9 |
|  |  | Strangulated hernia and small bowel obstruction |  |  |  |  | 3 | 0 | 0 | 3 |
|  | Hernia repair and pericardiocentesis |  |  |  | 1 | 0.5 |  |  |  |  |
|  |  | Strangulated hernia and pericardial effusion |  |  |  |  | 1 | 0 | 0 | 1 |
|  | Incision and drainage |  |  |  | 2 | 1.0 |  |  |  |  |
|  |  | Perianal abscess |  |  |  |  | 2 | 0 | 0 | 2 |
|  | Laparoscopy |  |  |  | 3 | 1.5 |  |  |  |  |
|  |  | Diagnostic for possible perforation |  |  |  |  | 1 | 0 | 0 | 1 |
|  |  | Small bowel obstruction |  |  |  |  | 1 | 0 | 0 | 1 |
|  |  | Washout post-appendicectomy |  |  |  |  | 1 | 0 | 0 | 1 |
|  | Laparotomy |  |  |  | 2 | 1.0 |  |  |  |  |
|  |  | Peritonitis |  |  |  |  | 1 | 0 | 0 | 1 |
|  |  | Unknown |  |  |  |  | 1 | 0 | 0 | 1 |
|  | Laparotomy - division of adhesions |  |  |  | 2 | 1.0 |  |  |  |  |
|  |  | Small bowel obstruction |  |  |  |  | 1 | 0 | 0 | 1 |
|  |  | Small bowel obstruction and choleduodenal fistula |  |  |  |  | 1 | 0 | 0 | 1 |
|  | Laparotomy - removal of gallstone ileus and hernia repair |  |  |  | 1 | 0.5 |  |  |  |  |
|  |  | Small bowel obstruction and choleduodenal fistula |  |  |  |  | 1 | 0 | 0 | 1 |
|  | Rectal examination under anesthesia |  |  |  | 1 | 0.5 |  |  |  |  |
|  |  | Bleeding from previous excision of rectal prolapse |  |  |  |  | 1 | 0 | 0 | 1 |
|  | Repair of duodenal perforation and washout |  |  |  | 1 | 0.5 |  |  |  |  |
|  |  | Duodenal perforation |  |  |  |  | 1 | 0 | 0 | 1 |
| **Cardiac** |  |  | 13 | 6.6 |  |  |  |  |  |  |
|  | BiVAD insertion |  |  |  | 1 | 0.5 |  |  |  |  |
|  |  | BiVAD insertion |  |  |  |  | 1 | 0 | 0 | 1 |
|  | CABG |  |  |  | 1 | 0.5 |  |  |  |  |
|  |  | NSTEMI and shock |  |  |  |  | 1 | 0 | 0 | 1 |
|  | Drain - pericardial drain |  |  |  | 7 | 3.5 |  |  |  |  |
|  |  | Pericardial effusion |  |  |  |  | 5 | 0 | 1 | 4 |
|  |  | Pericardial effusion and tamponade |  |  |  |  | 2 | 0 | 0 | 2 |
|  | Pacemaker insertion |  |  |  | 4 | 2.0 |  |  |  |  |
|  |  | Collapse |  |  |  |  | 1 | 0 | 0 | 1 |
|  |  | Complete heart block |  |  |  |  | 2 | 0 | 0 | 2 |
|  |  | Pauses |  |  |  |  | 1 | 0 | 0 | 1 |
| **Genitourinary** |  |  | 18 | 9.1 |  |  |  |  |  |  |
|  | Cystoscopy |  |  |  | 1 | 0.5 |  |  |  |  |
|  |  | Cystoscopy |  |  |  |  | 1 | 0 | 0 | 1 |
|  | Cystoscopy + stent insertion |  |  |  | 1 | 0.5 |  |  |  |  |
|  |  | Obstructive nephropathy |  |  |  |  | 1 | 0 | 0 | 1 |
|  | Nephrostomy |  |  |  | 10 | 5.1 |  |  |  |  |
|  |  | Obstructive nephropathy |  |  |  |  | 9 | 0 | 0 | 9 |
|  |  | Perinephric abscess |  |  |  |  | 1 | 0 | 0 | 1 |
|  | Orchidectomy |  |  |  | 1 | 0.5 |  |  |  |  |
|  |  | Necrotizing scrotal abscess |  |  |  |  | 1 | 0 | 0 | 1 |
|  | Stent - ureter |  |  |  | 2 | 1.0 |  |  |  |  |
|  |  | Obstructive nephropathy |  |  |  |  | 2 | 0 | 0 | 2 |
|  | Suprapubic catheter insertion |  |  |  | 3 | 1.5 |  |  |  |  |
|  |  | Urinary retention |  |  |  |  | 3 | 0 | 0 | 3 |
| **Gynecology** |  |  | 2 | 1.0 |  |  |  |  |  |  |
|  | Removal of retained products of conception |  |  |  | 1 | 0.5 |  |  |  |  |
|  |  | Retained products of conception |  |  |  |  | 1 | 0 | 0 | 1 |
|  | Salpingectomy |  |  |  | 1 | 0.5 |  |  |  |  |
|  |  | Ectopic pregnancy |  |  |  |  | 1 | 0 | 0 | 1 |
| **Neck** |  |  | 2 | 1.0 |  |  |  |  |  |  |
|  | Drain - neck abscess |  |  |  | 1 | 0.5 |  |  |  |  |
|  |  | Ludwig angina secondary to peritonsillar abscess |  |  |  |  | 1 | 0 | 0 | 1 |
|  | Tracheostomy |  |  |  | 1 | 0.5 |  |  |  |  |
|  |  | Laryngeal obstruction |  |  |  |  | 1 | 0 | 0 | 1 |
| **Neurosurgery** |  |  | 4 | 2.0 |  |  |  |  |  |  |
|  | Laminectomy |  |  |  | 1 | 0.5 |  |  |  |  |
|  |  | Cauda equina syndrome |  |  |  |  | 1 | 0 | 0 | 1 |
|  | Spinal surgery |  |  |  | 2 | 1.0 |  |  |  |  |
|  |  | Cauda equina syndrome |  |  |  |  | 1 | 0 | 0 | 1 |
|  |  | Cervical spine surgery for traumatic fracture and cord injury |  |  |  |  | 1 | 0 | 0 | 1 |
|  | VP shunt |  |  |  | 1 | 0.5 |  |  |  |  |
|  |  | Hydrocephalus |  |  |  |  | 1 | 0 | 1 | 0 |
| **Ophthalmology** |  |  | 1 | 0.5 |  |  |  |  |  |  |
|  | Orbital decompression |  |  |  | 1 | 0.5 |  |  |  |  |
|  |  | Progressive facial swelling and diplopia |  |  |  |  | 1 | 0 | 0 | 1 |
| **Orthopedic** |  |  | 21 | 10.6 |  |  |  |  |  |  |
|  | Fractured femur + right forearm repair |  |  |  | 1 | 0.5 |  |  |  |  |
|  |  | Fractured femur and forearm |  |  |  |  | 1 | 0 | 0 | 1 |
|  | Fractured femur repair |  |  |  | 3 | 1.5 |  |  |  |  |
|  |  | Fractured femur |  |  |  |  | 3 | 0 | 0 | 3 |
|  | Fractured neck of femur repair |  |  |  | 11 | 5.6 |  |  |  |  |
|  |  | Fractured neck of femur |  |  |  |  | 11 | 0 | 3 | 8 |
|  | Fractured pelvis |  |  |  | 1 | 0.5 |  |  |  |  |
|  |  | Fractured pelvis |  |  |  |  | 1 | 0 | 0 | 1 |
|  | Fractured tibia repair |  |  |  | 1 | 0.5 |  |  |  |  |
|  |  | Fractured tibia |  |  |  |  | 1 | 0 | 0 | 1 |
|  | Knee replacement revision |  |  |  | 1 | 0.5 |  |  |  |  |
|  |  | Bleeding from knee wound |  |  |  |  | 1 | 0 | 0 | 1 |
|  | Washout - knee |  |  |  | 3 | 1.5 |  |  |  |  |
|  |  | Septic arthritis |  |  |  |  | 2 | 0 | 0 | 2 |
|  |  | Septic arthritis (knee replacement) |  |  |  |  | 1 | 0 | 0 | 1 |
| **Skin** |  |  | 9 | 4.5 |  |  |  |  |  |  |
|  | Skin debridement |  |  |  | 6 | 3.0 |  |  |  |  |
|  |  | Necrotizing fasciitis |  |  |  |  | 2 | 1 | 0 | 1 |
|  |  | Fournier gangrene |  |  |  |  | 3 | 0 | 0 | 3 |
|  |  | Infected fat necrosis |  |  |  |  | 1 | 0 | 0 | 1 |
|  | Debridement - face |  |  |  | 1 | 0.5 |  |  |  |  |
|  |  | Retrobulbar infection |  |  |  |  | 1 | 0 | 0 | 1 |
|  | Debridement - pseudoaneurysm |  |  |  | 1 | 0.5 |  |  |  |  |
|  |  | Iliopsoas abscess and pseudoaneurysm |  |  |  |  | 1 | 0 | 0 | 1 |
|  | Skin - evacuation of infected haematoma |  |  |  | 1 | 0.5 |  |  |  |  |
|  |  | Infected haematoma |  |  |  |  | 1 | 0 | 0 | 1 |
| **Thoracic** |  |  | 22 | 11.1 |  |  |  |  |  |  |
|  | Chest drain |  |  |  | 19 | 9.6 |  |  |  |  |
|  |  | Hemothorax |  |  |  |  | 3 | 1 | 1 | 1 |
|  |  | Pleural effusion |  |  |  |  | 9 | 0 | 0 | 9 |
|  |  | Pneumothorax |  |  |  |  | 7 | 0 | 2 | 5 |
|  | Drain - chest - CT guided |  |  |  | 1 | 0.5 |  |  |  |  |
|  |  | Bullae |  |  |  |  | 1 | 0 | 0 | 1 |
|  | Drain - chest - open |  |  |  | 1 | 0.5 |  |  |  |  |
|  |  | Hemopneumothorax |  |  |  |  | 1 | 0 | 0 | 1 |
|  | VATS + ICD insertion |  |  |  | 1 | 0.5 |  |  |  |  |
|  |  | Pleural effusion |  |  |  |  | 1 | 0 | 0 | 1 |
| **Unknown** |  |  | 5 | 2.5 |  |  |  |  |  |  |
|  | Unknown |  |  |  | 4 | 2.0 |  |  |  |  |
|  |  | Unknown |  |  |  |  | 4 | 0 | 4 | 0 |
|  | Washout - wound |  |  |  | 1 | 0.5 |  |  |  |  |
|  |  | Unknown |  |  |  |  | 1 | 0 | 1 | 0 |
| **Vascular** |  |  | 10 | 5.1 |  |  |  |  |  |  |
|  | Aortic arch replacement |  |  |  | 1 | 0.5 |  |  |  |  |
|  |  | Aortic dissection |  |  |  |  | 1 | 0 | 0 | 1 |
|  | Aortic dissection repair |  |  |  | 2 | 1.0 |  |  |  |  |
|  |  | Aortic dissection |  |  |  |  | 2 | 1 | 0 | 1 |
|  | CVC insertion |  |  |  | 4 | 2.0 |  |  |  |  |
|  |  | Inotropic support |  |  |  |  | 3 | 0 | 0 | 3 |
|  |  | Renal replacement therapy |  |  |  |  | 1 | 0 | 0 | 1 |
|  | CVC removal |  |  |  | 1 | 0.5 |  |  |  |  |
|  |  | Line sepsis |  |  |  |  | 1 | 0 | 0 | 1 |
|  | Embolization of splenic artery pseudoaneurysm |  |  |  | 1 | 0.5 |  |  |  |  |
|  |  | Splenic artery pseudoaneurysm |  |  |  |  | 1 | 0 | 0 | 1 |
|  | Femoral pseudoaneurysm - exploration |  |  |  | 1 | 0.5 |  |  |  |  |
|  |  | Infected and bleeding left groin pseudoaneurysm |  |  |  |  | 1 | 0 | 0 | 1 |

**Supplementary Table 2. Summary of key data points for each patient included in analysis.**

| Age | Sex | Active cancer? | Direct oral anticoagulant | Procedural specialty | Procedure | Indication for procedure | Procedural bleeding risk | Creatinine (μmol/L) | Time from reversal to procedure (hours) | Time from last dose of DOAC to reversal (hours) | Red cell units transfused in 48 hours post-procedure | 90-day mortality | Days to death |
| --- | --- | --- | --- | --- | --- | --- | --- | --- | --- | --- | --- | --- | --- |
| 71 | M |  | Rivaroxaban | Abdominal | Appendicectomy | Appendicitis | Low-moderate | 92 | 9.0 |  |  | No |  |
| 66 | M |  | Rivaroxaban | Abdominal | Appendicectomy | Appendicitis | Low-moderate | 95 | 1.5 | 27.8 |  | No |  |
| 84 | M |  | Rivaroxaban | Abdominal | Appendicectomy | Appendicitis - perforated | Low-moderate | 113 | 4.5 | 9.3 |  | No |  |
| 75 | F |  | Apixaban | Abdominal | Appendectomy (laparotomy) | Appendicitis | Low-moderate | 59 | 2.5 |  |  | No |  |
| 68 | M |  | Rivaroxaban | Abdominal | Appendectomy (laparotomy) | Appendicitis - perforated | Low-moderate | 135 |  |  |  | Yes | 30 |
| 78 | M |  | Apixaban | Abdominal | Appendectomy (laparoscopy) | Appendicitis | Low-moderate | 108 | 0.4 | 22.3 |  | No |  |
| 83 | F |  | Apixaban | Abdominal | Appendectomy (laparoscopy) | Appendicitis | Low-moderate | 124 | 0.8 |  |  | No |  |
| 52 | F |  | Apixaban | Abdominal | Appendectomy (laparoscopy) | Appendicitis | Low-moderate | 114 |  |  |  | No |  |
| 69 | M |  | Apixaban | Abdominal | Appendectomy (laparoscopy) | Appendicitis - perforated | Low-moderate | 174 |  |  |  | No |  |
| 70 | F |  | Dabigatran | Abdominal | Bowel resection | Bowel ischemia | High | 108 |  |  |  | Yes | 1 |
| 80 | F |  | Apixaban | Abdominal | Bowel resection | Bowel ischemia | High | 93 | 1.4 |  |  | No |  |
| 91 | M |  | Apixaban | Abdominal | Bowel resection | Bowel ischemia | High | 138 |  |  |  | No |  |
| 68 | F |  | Apixaban | Abdominal | Bowel resection | Bowel ischemia | High | 35 |  |  |  | Yes | 4 |
| 71 | M | Colorectal | Apixaban | Abdominal | Bowel resection | Bowel obstruction | High | 79 |  |  |  | No |  |
| 79 | M |  | Apixaban | Abdominal | Bowel resection | Bowel obstruction | High | 72 |  |  |  | No |  |
| 67 | M | Colorectal | Edoxaban | Abdominal | Bowel resection | Bowel obstruction | High | 75 | 0.5 |  |  | No |  |
| 75 | F |  | Apixaban | Abdominal | Bowel resection | Bowel obstruction | High | 353 |  |  |  | No |  |
| 82 | M |  | Rivaroxaban | Abdominal | Bowel resection | Bowel obstruction | High | 75 | 0.6 | 34.5 | 4 | No |  |
| 63 | M |  | Apixaban | Abdominal | Bowel resection | Bowel obstruction and appendicitis | High | 86 | 0.2 |  |  | No |  |
| 86 | M |  | Edoxaban | Abdominal | Bowel resection | Bowel perforation | High | 221 |  |  |  | Yes | 10 |
| 71 | F | Colorectal | Apixaban | Abdominal | Bowel resection | Large bowel obstruction | High | 50 | 0.2 |  | 2 | No |  |
| 68 | M | Prostate | Edoxaban | Abdominal | Bowel resection | Large bowel obstruction | High | 123 | 13.2 |  |  | No |  |
| 72 | F |  | Rivaroxaban | Abdominal | Bowel resection | Large bowel obstruction | High | 59 |  |  | 2 | No |  |
| 77 | M | Multiple myeloma | Apixaban | Abdominal | Bowel resection | Large bowel perforation | High | 183 |  |  |  | No |  |
| 88 | F |  | Apixaban | Abdominal | Bowel resection | Large bowel perforation | High | 91 | 1.2 | 13.1 |  | No |  |
| 78 | M | Colorectal | Edoxaban | Abdominal | Bowel resection | Large bowel perforation - impending | High | 118 |  |  |  | No |  |
| 84 | F |  | Apixaban | Abdominal | Bowel resection | Rectal prolapse | High | 90 | 2.4 | 17.0 |  | No |  |
| 83 | F |  | Edoxaban | Abdominal | Bowel resection | Sigmoid volvulus | High | 44 |  |  |  | No |  |
| 83 | M |  | Apixaban | Abdominal | Bowel resection | Small bowel obstruction | High | 111 | 0.2 | 29.4 |  | No |  |
| 92 | F |  | Apixaban | Abdominal | Bowel resection | Small bowel obstruction | High | 52 |  |  |  | No |  |
| 73 | F |  | Apixaban | Abdominal | Bowel resection | Small bowel obstruction | High | 60 |  |  |  | No |  |
| 40 | M |  | Rivaroxaban | Abdominal | Bowel resection | Small bowel obstruction | High | 23 |  |  |  | No |  |
| 77 | M |  | Apixaban | Abdominal | Bowel resection | Small bowel obstruction | High | 96 |  |  |  | No |  |
| 85 | M |  | Apixaban | Abdominal | Bowel resection | Strangulated femoral hernia | High | 80 |  |  |  | No |  |
| 79 | M |  | Apixaban | Abdominal | Bowel resection | Unknown | High | 104 | 4.5 |  |  | No |  |
| 73 | M | Glioblastoma | Apixaban | Abdominal | Bowel resection | Unknown | High | 118 | 1.5 | 26.8 |  | Yes | 32 |
| 76 | F |  | Apixaban | Abdominal | Bowel resection | Ventral hernia | High | 79 |  | 22.7 |  | No |  |
| 71 | F |  | Rivaroxaban | Abdominal | Bowel resection + lower limb embolectomy | Bowel perforation and ischemic leg | High | 185 |  |  | 2 | No |  |
| 57 | M | Colorectal | Apixaban | Abdominal | Bowel resection | Large bowel obstruction | High | 83 | 1.5 |  |  | No |  |
| 82 | M |  | Apixaban | Abdominal | Cholecystectomy | Cholecystitis | Low-moderate | 153 | 4.5 | 22.6 |  | No |  |
| 82 | M |  | Rivaroxaban | Abdominal | Cholecystectomy | Cholecystitis | Low-moderate | 74 | 1.5 |  | 2 | No |  |
| 66 | M |  | Apixaban | Abdominal | Drain - ascitic | Ascites | Minimal | 330 |  |  |  | Yes | 30 |
| 52 | M |  | Apixaban | Abdominal | Drain - ascitic | Ascites | Minimal | 66 |  |  |  | No |  |
| 79 | F | Breast | Apixaban | Abdominal | Drain - ascitic | Ascites | Minimal | 106 |  |  |  | Yes | 75 |
| 73 | M | Colorectal | Rivaroxaban | Abdominal | Drain - intrabdominal abscess | Intrabdominal abscess | High | 51 |  | 27.2 |  | No |  |
| 38 | F | Cholangiocarcinoma | Rivaroxaban | Abdominal | Drain - liver abscess | Liver abscess | High | 49 |  |  |  | Yes | 47 |
| 62 | M |  | Apixaban | Abdominal | Drain - liver abscess | Liver abscess | High | 918 |  | 32.0 |  | No |  |
| 85 | F |  | Apixaban | Abdominal | Hernia - repair | Hernia | Low-moderate | 70 | 1.5 |  |  | No |  |
| 80 | F |  | Apixaban | Abdominal | Hernia repair | Incarcerated hernia | Low-moderate | 75 | 1.5 | 8.8 |  | No |  |
| 83 | F |  | Apixaban | Abdominal | Hernia repair | Incarcerated hernia | Low-moderate | 59 | 1.5 | 12.3 |  | No |  |
| 81 | M |  | Rivaroxaban | Abdominal | Hernia repair | Incarcerated hernia | Low-moderate | 76 |  |  |  | No |  |
| 81 | F |  | Apixaban | Abdominal | Hernia repair | Incarcerated hernia | Low-moderate | 125 |  |  |  | No |  |
| 50 | F |  | Apixaban | Abdominal | Hernia repair | Incarcerated hernia | Low-moderate | 74 |  |  |  | No |  |
| 78 | F |  | Apixaban | Abdominal | Hernia repair | Incarcerated hernia | Low-moderate | 71 |  | 33.5 |  | No |  |
| 74 | F |  | Rivaroxaban | Abdominal | Hernia repair | Incarcerated hernia | Low-moderate | 92 |  |  |  | No |  |
| 75 | M |  | Apixaban | Abdominal | Hernia repair | Incarcerated hernia | Low-moderate | 110 |  |  |  | No |  |
| 81 | M |  | Apixaban | Abdominal | Hernia repair | Small bowel obstruction | Low-moderate | 80 |  | 24.0 |  | No |  |
| 84 | M |  | Apixaban | Abdominal | Hernia repair | Small bowel obstruction | Low-moderate | 89 | 24.5 | 7.7 |  | Yes | 4 |
| 87 | M |  | Edoxaban | Abdominal | Hernia repair | Small bowel obstruction | Low-moderate | 77 |  |  |  | No |  |
| 88 | F |  | Rivaroxaban | Abdominal | Hernia repair | Small bowel obstruction | Low-moderate | 109 |  |  |  | No |  |
| 74 | F |  | Edoxaban | Abdominal | Hernia repair | Strangulated hernia | Low-moderate | 61 |  |  |  | No |  |
| 56 | M |  | Apixaban | Abdominal | Hernia repair | Strangulated hernia | Low-moderate | 82 | 5.8 | 21.2 |  | No |  |
| 78 | M |  | Edoxaban | Abdominal | Hernia repair | Strangulated hernia | Low-moderate | 146 |  |  |  | No |  |
| 78 | M |  | Edoxaban | Abdominal | Hernia repair | Strangulated hernia | Low-moderate | 139 |  |  |  | No |  |
| 78 | M | Multiple myeloma | Apixaban | Abdominal | Hernia repair | Strangulated hernia | Low-moderate | 121 |  | 23.7 |  | No |  |
| 78 | F |  | Apixaban | Abdominal | Hernia repair | Strangulated hernia | Low-moderate | 40 |  |  |  | No |  |
| 74 | F | Melanoma | Apixaban | Abdominal | Hernia repair | Strangulated hernia | Low-moderate | 88 | 3.9 | 9.1 |  | No |  |
| 81 | F |  | Apixaban | Abdominal | Hernia repair | Strangulated hernia | Low-moderate | 69 | 8.0 | 24.1 |  | No |  |
| 92 | M |  | Rivaroxaban | Abdominal | Hernia repair | Strangulated hernia and small bowel obstruction | Low-moderate | 129 |  |  |  | No |  |
| 78 | M | Skin | Edoxaban | Abdominal | Hernia repair | Strangulated hernia and small bowel obstruction | Low-moderate | 85 |  |  |  | No |  |
| 77 | F |  | Apixaban | Abdominal | Hernia repair | Strangulated hernia and small bowel obstruction | Low-moderate | 44 |  |  |  | No |  |
| 77 | M |  | Dabigatran | Abdominal | Hernia repair - open | Incarcerated hernia | Low-moderate | 98 | 1.8 |  |  | No |  |
| 71 | F |  | Dabigatran | Abdominal | Hernia repair - open | Incarcerated hernia | Low-moderate | 56 | 1.7 |  |  | No |  |
| 87 | F |  | Apixaban | Abdominal | Hernia repair - open | Incarcerated hernia | Low-moderate | 74 |  |  |  | No |  |
| 81 | M |  | Edoxaban | Abdominal | Hernia repair - open | Incarcerated hernia | Low-moderate | 128 |  |  |  | No |  |
| 86 | M |  | Apixaban | Abdominal | Hernia repair - open | Incarcerated hernia and small bowel obstruction | Low-moderate | 76 |  |  |  | No |  |
| 84 | F |  | Dabigatran | Abdominal | Hernia repair - open | Small bowel obstruction | Low-moderate | 77 | 2.0 |  |  | No |  |
| 90 | F |  | Apixaban | Abdominal | Hernia repair - open | Strangulated hernia | Low-moderate | 106 |  |  |  | No |  |
| 37 | M |  | Rivaroxaban | Abdominal | Hernia repair and pericardiocentesis | Strangulated hernia and pericardial effusion | Low-moderate | 40 |  |  |  | No |  |
| 60 | M |  | Apixaban | Abdominal | Incision and drainage | Perianal abscess | Low-moderate | 165 |  |  |  | No |  |
| 77 | M |  | Rivaroxaban | Abdominal | Incision and drainage | Perianal abscess | Low-moderate | 48 | 3.4 |  |  | No |  |
| 75 | F |  | Rivaroxaban | Abdominal | Laparoscopy | Diagnostic for possible perforation | Low-moderate | 81 |  |  |  | No |  |
| 77 | M | Colorectal | Rivaroxaban | Abdominal | Laparoscopy | Small bowel obstruction | Low-moderate | 147 |  |  |  | No |  |
| 52 | M |  | Apixaban | Abdominal | Laparoscopy | Washout post-appendicectomy | Low-moderate | 75 |  | 10.6 |  | No |  |
| 50 | F |  | Apixaban | Abdominal | Laparotomy | Peritonitis | High | 63 |  |  |  | Yes | 60 |
| 80 | M |  | Rivaroxaban | Abdominal | Laparotomy | Unknown | High | 211 | 1.5 |  |  | No |  |
| 65 | F |  | Apixaban | Abdominal | Laparotomy - division of adhesions | Small bowel obstruction | High | 90 | 1.0 |  |  | No |  |
| 88 | M |  | Edoxaban | Abdominal | Laparotomy - division of adhesions | Small bowel obstruction | High | 106 |  |  |  | No |  |
| 63 | F |  | Apixaban | Abdominal | Laparotomy - removal of gallstone ileus and hernia repair | Small bowel obstruction and choleduodenal fistula | High | 0 |  |  |  | No |  |
| 66 | F |  | Edoxaban | Abdominal | Rectal examination under anesthesia | Bleeding from previous excision of rectal prolapse | Minimal | 45 | 0.0 | 7.0 | 1 | No |  |
| 69 | M |  | Apixaban | Abdominal | Repair of duodenal perforation and washout | Duodenal perforation | Low-moderate | 78 | 0.0 |  | 1 | No |  |
| 53 | M |  | Apixaban | Cardiac | BiVAD insertion | BiVAD insertion | Minimal | 126 | 4.2 |  | 2 | Yes | 26 |
| 71 | M |  | Edoxaban | Cardiac | CABG | NSTEMI and shock | High | 104 |  |  |  | No |  |
| 80 | F |  | Rivaroxaban | Cardiac | Drain - pericardial drain | Pericardial effusion | High | 288 |  | 25.0 |  | Yes | 3 |
| 64 | M |  | Dabigatran | Cardiac | Drain - pericardial drain | Pericardial effusion | High | 107 |  | 3.9 |  | No |  |
| 78 | F |  | Edoxaban | Cardiac | Drain - pericardial drain | Pericardial effusion | High | 211 | 1.2 | 15.3 |  | No |  |
| 40 | M |  | Apixaban | Cardiac | Drain - pericardial drain | Pericardial effusion | High | 415 |  |  | 1 | No |  |
| 66 | M |  | Apixaban | Cardiac | Drain - pericardial drain | Pericardial effusion | High | 169 |  |  |  | No |  |
| 85 | M |  | Edoxaban | Cardiac | Drain - pericardial drain | Pericardial effusion and tamponade | High | 66 |  |  | 1 | Yes | 34 |
| 78 | M |  | Apixaban | Cardiac | Drain - pericardial drain | Pericardial effusion and tamponade | High | 493 |  | 31.2 |  | Yes | 17 |
| 89 | M |  | Apixaban | Cardiac | Pacemaker insertion | Collapse | Minimal | 143 | 1.8 | 7.2 |  | No |  |
| 57 | M |  | Edoxaban | Cardiac | Pacemaker insertion | Complete heart block | Minimal | 76 |  |  |  | No |  |
| 78 | F |  | Rivaroxaban | Cardiac | Pacemaker insertion | Complete heart block | Minimal | 199 |  |  |  | No |  |
| 78 | M |  | Rivaroxaban | Cardiac | Pacemaker insertion | Pauses | Minimal | 143 |  |  |  | No |  |
| 79 | M | Bladder | Rivaroxaban | Genitourinary | Cystoscopy | Cystoscopy | Minimal | 167 |  |  | 1 | Yes | 47 |
| 86 | M | Prostate | Apixaban | Genitourinary | Cystoscopy + stent insertion | Obstructive nephropathy | Low-moderate | 733 |  |  |  | Yes | 39 |
| 83 | F | Breast | Edoxaban | Genitourinary | Nephrostomy | Obstructive nephropathy | High | 209 |  |  |  | No |  |
| 78 | M | Colorectal | Apixaban | Genitourinary | Nephrostomy | Obstructive nephropathy | High | 617 | 2.0 |  |  | No |  |
| 73 | M |  | Apixaban | Genitourinary | Nephrostomy | Obstructive nephropathy | High | 139 |  |  |  | No |  |
| 74 | M |  | Apixaban | Genitourinary | Nephrostomy | Obstructive nephropathy | High | 192 |  |  |  | No |  |
| 80 | F | Bladder | Apixaban | Genitourinary | Nephrostomy | Obstructive nephropathy | High | 162 | 0.5 | 45.0 |  | No |  |
| 85 | F |  | Apixaban | Genitourinary | Nephrostomy | Obstructive nephropathy | High | 213 | 93.9 |  |  | No |  |
| 53 | M |  | Apixaban | Genitourinary | Nephrostomy | Obstructive nephropathy | High | 99 |  |  |  | Yes | 85 |
| 76 | F |  | Apixaban | Genitourinary | Nephrostomy | Obstructive nephropathy | High | 189 |  |  |  | No |  |
| 45 | F |  | Rivaroxaban | Genitourinary | Nephrostomy | Perinephric abscess | High | 121 | 2.3 | 38.0 |  | No |  |
| 75 | M |  | Apixaban | Genitourinary | Orchidectomy | Necrotizing scrotal abscess | Low-moderate | 180 |  |  |  | No |  |
| 75 | M | Esophageal/stomach | Rivaroxaban | Genitourinary | Stent - ureter | Obstructive nephropathy | Low-moderate |  |  | 28.5 |  | Yes | 24 |
| 63 | M | Prostate | Rivaroxaban | Genitourinary | Stent - ureter | Obstructive nephropathy | Low-moderate | 593 |  |  |  | Yes | 76 |
| 86 | M |  | Apixaban | Genitourinary | Suprapubic catheter insertion | Urinary retention | Low-moderate | 100 |  |  |  | No |  |
| 79 | M |  | Apixaban | Genitourinary | Suprapubic catheter insertion | Urinary retention | Low-moderate | 91 |  |  |  | No |  |
| 78 | F | Paget's disease (extra-mammary) | Apixaban | Genitourinary | Suprapubic catheter insertion | Urinary retention | Low-moderate | 108 | 2.4 | 11.8 |  | Yes | 62 |
| 75 | F |  | Rivaroxaban | Genitourinary | Ureteric obstruction | Obstructive nephropathy | Low-moderate | 218 | 1.4 | 23.3 |  | No |  |
| 28 | F |  | Apixaban | Gynecology | Removal of retained products of conception | Retained products of conception | High | 58 |  |  | 2 | No |  |
| 38 | F |  | Apixaban | Gynecology | Salpingectomy | Ectopic pregnancy | High | 66 |  |  |  | No |  |
| 66 | F |  | Apixaban | Neck | Drain - neck abscess | Ludwig angina secondary to peritonsillar abscess | High | 65 |  |  |  | No |  |
| 85 | M |  | Apixaban | Neck | Tracheostomy | Laryngeal obstruction | Low-moderate | 103 |  |  |  | No |  |
| 51 | M |  | Rivaroxaban | Neurosurgery | Laminectomy | Cauda equina syndrome | High | 129 | 5.7 | 20.5 |  | No |  |
| 41 | M |  | Apixaban | Neurosurgery | Spinal surgery | Cauda equina syndrome | High | 92 |  |  |  | No |  |
| 89 | M |  | Apixaban | Neurosurgery | Spinal surgery | Cervical spine surgery for traumatic fracture and cord injury (not actually performed) | High | 148 |  |  |  | Yes | 1 |
| 30 | M |  | Dabigatran | Neurosurgery | VP shunt | Hydrocephalus | High | 59 |  |  |  | No |  |
| 65 | M | Orbital/sinonasal lymphoma | Apixaban | Ophthalmology | Orbital decompression | Progressive facial swelling and diplopia | High | 50 |  |  |  | No |  |
| 88 | M |  | Apixaban | Orthopedic | Fractured femur + right forearm repair | Fractured femur and forearm | High | 72 |  |  |  | No |  |
| 91 | M |  | Apixaban | Orthopedic | Fractured femur repair | Fractured femur | High | 69 |  |  |  | No |  |
| 86 | M |  | Apixaban | Orthopedic | Fractured femur repair | Fractured femur | High | 123 |  |  | 1 | Yes | 6 |
| 78 | M | Urothelial | Rivaroxaban | Orthopedic | Fractured femur repair | Fractured femur | High | 84 |  |  |  | Yes | 90 |
| 99 | M |  | Dabigatran | Orthopedic | Fractured neck of femur repair | Fractured neck of femur | High | 111 | 9.0 |  |  | No |  |
| 92 | F |  | Dabigatran | Orthopedic | Fractured neck of femur repair | Fractured neck of femur | High | 88 |  |  | 1 | Yes | 1 |
| 99 | F |  | Apixaban | Orthopedic | Fractured neck of femur repair | Fractured neck of femur | High | 153 | 3.0 | 48.0 |  | Yes | 22 |
| 78 | F |  | Rivaroxaban | Orthopedic | Fractured neck of femur repair | Fractured neck of femur | High | 62 | 27.4 |  | 2 | No |  |
| 74 | M | Prostate | Apixaban | Orthopedic | Fractured neck of femur repair | Fractured neck of femur | High | 47 |  |  | 1 | Yes | 20 |
| 86 | F |  | Dabigatran | Orthopedic | Fractured neck of femur repair | Fractured neck of femur | High | 56 |  |  |  | No |  |
| 76 | M | Esophageal | Edoxaban | Orthopedic | Fractured neck of femur repair | Fractured neck of femur | High | 168 | 2.2 | 29.8 |  | No |  |
| 90 | F |  | Edoxaban | Orthopedic | Fractured neck of femur repair | Fractured neck of femur | High | 105 |  |  |  | No |  |
| 86 | M |  | Rivaroxaban | Orthopedic | Fractured neck of femur repair | Fractured neck of femur | High | 98 | 1.4 | 42.6 |  | No |  |
| 98 | M |  | Apixaban | Orthopedic | Fractured neck of femur repair | Fractured neck of femur | High | 253 | 4.3 |  | 1 | Yes | 21 |
| 96 | F |  | Rivaroxaban | Orthopedic | Fractured neck of femur repair | Fractured neck of femur | High | 97 | 6.9 |  | 1 | Yes | 24 |
| 81 | F |  | Apixaban | Orthopedic | Fractured pelvis | Fractured pelvis | High | 112 |  |  | 2 | Yes | 40 |
| 70 | M |  | Rivaroxaban | Orthopedic | Fractured tibia repair | Fractured tibia | High | 56 | 5.7 |  |  | No |  |
| 67 | M |  | Rivaroxaban | Orthopedic | Knee replacement revision | Bleeding from knee wound | High | 74 |  |  |  | No |  |
| 89 | F |  | Apixaban | Orthopedic | Washout - knee | Septic arthritis | Low-moderate | 142 | 0.3 |  |  | Yes | 13 |
| 85 | F |  | Rivaroxaban | Orthopedic | Washout - knee | Septic arthritis | Low-moderate | 178 |  |  |  | Yes | 6 |
| 92 | M | Prostate | Apixaban | Orthopedic | Washout - knee | Septic arthritis (knee replacement) | Low-moderate | 84 |  |  |  | No |  |
| 73 | M |  | Rivaroxaban | Skin | Debridement | Necrotizing fasciitis | High | 96 |  |  | 4 | No |  |
| 73 | M |  | Rivaroxaban | Skin | Debridement - face | Retrobulbar infection | High | 158 |  |  |  | No |  |
| 35 | M |  | Apixaban | Skin | Debridement - pseudoaneurysm | Iliopsoas abscess and pseudoaneurysm | High | 110 |  |  |  | No |  |
| 82 | M | Prostate | Apixaban | Skin | Skin - debridement | Fournier gangrene | High | 128 | 0.6 | 4.8 | 2 | Yes | 8 |
| 56 | M |  | Edoxaban | Skin | Skin - debridement | Fournier gangrene | High | 78 |  |  | 7 | No |  |
| 45 | M |  | Rivaroxaban | Skin | Skin - debridement | Fournier gangrene | High | 99 |  |  |  | No |  |
| 75 | F |  | Apixaban | Skin | Skin - debridement | Infected fat necrosis | High | 114 | 0.5 | 13.5 | 1 | No |  |
| 79 | F |  | Apixaban | Skin | Skin - debridement | Necrotizing fasciitis | High | 172 | 0.5 |  | 5 | Yes | 35 |
| 86 | F |  | Edoxaban | Skin | Skin - evacuation of infected haematoma | Infected haematoma | High | 103 | 0.3 |  |  | Yes | 41 |
| 84 | M |  | Dabigatran | Thoracic | Drain - chest | Hemothorax | Minimal | 112 | 3.4 |  | 1 | No |  |
| 79 | F |  | Rivaroxaban | Thoracic | Drain - chest | Hemothorax | Minimal | 121 | 2.1 |  | 3 | No |  |
| 85 | F |  | Rivaroxaban | Thoracic | Drain - chest | Hemothorax | Minimal | 110 |  | 28.8 |  | Yes | 1 |
| 43 | M |  | Apixaban | Thoracic | Drain - chest | Pleural effusion | Minimal | 102 | 1.5 |  |  | No |  |
| 74 | M | Mesothelioma | Rivaroxaban | Thoracic | Drain - chest | Pleural effusion | Minimal | 87 |  |  |  | No |  |
| 81 | F |  | Apixaban | Thoracic | Drain - chest | Pleural effusion | Minimal | 142 | 1.3 |  |  | Yes | 90 |
| 86 | M |  | Rivaroxaban | Thoracic | Drain - chest | Pleural effusion | Minimal | 152 |  |  |  | No |  |
| 78 | F | Lung | Edoxaban | Thoracic | Drain - chest | Pleural effusion | Minimal | 66 |  |  |  | Yes | 8 |
| 85 | F |  | Apixaban | Thoracic | Drain - chest | Pleural effusion | Minimal | 160 |  | 33.0 |  | Yes | 4 |
| 78 | M | Lung | Apixaban | Thoracic | Drain - chest | Pleural effusion | Minimal | 81 |  |  |  | Yes | 54 |
| 54 | F | Gynecological | Apixaban | Thoracic | Drain - chest | Pleural effusion | Minimal | 56 |  |  |  | Yes | 41 |
| 81 | M |  | Dabigatran | Thoracic | Drain - chest | Pneumothorax | Minimal | 82 |  |  |  | No |  |
| 83 | F |  | Rivaroxaban | Thoracic | Drain - chest | Pneumothorax | Minimal | 41 | 0.0 |  |  | Yes | 29 |
| 79 | F |  | Apixaban | Thoracic | Drain - chest | Pneumothorax | Minimal | 73 |  |  |  | Yes | 30 |
| 61 | M |  | Dabigatran | Thoracic | Drain - chest | Pneumothorax | Minimal | 52 | 2.5 |  |  | No |  |
| 80 | M | JAK2+ MPN | Apixaban | Thoracic | Drain - chest | Pneumothorax | Minimal | 113 |  |  |  | No |  |
| 69 | M |  | Apixaban | Thoracic | Drain - chest | Pneumothorax | Minimal | 40 | 0.6 |  |  | No |  |
| 91 | M |  | Edoxaban | Thoracic | Drain - chest | Pneumothorax | Minimal | 125 |  |  |  | Yes | 10 |
| 78 | M |  | Apixaban | Thoracic | Drain - chest | Unknown | Minimal | 108 |  |  |  | No |  |
| 69 | M |  | Apixaban | Thoracic | Drain - chest - CT guided | Bullae | Low-moderate | 102 | 2.0 |  |  | No |  |
| 54 | M |  | Apixaban | Thoracic | Drain - chest - open | Hemopneumothorax | Minimal | 65 | 0.8 | 14.7 |  | No |  |
| 65 | M |  | Rivaroxaban | Thoracic | VATS + ICD insertion | Pleural effusion | Low-moderate | 61 |  |  |  | No |  |
| 79 | F |  | Dabigatran | Unknown | Unknown | Unknown | Unknown | 63 |  |  |  | No |  |
| 76 | F |  | Dabigatran | Unknown | Unknown | Unknown | Unknown | 71 |  |  |  | No |  |
| 90 | M |  | Dabigatran | Unknown | Unknown | Unknown | Unknown | 70 |  | 45.8 |  | Yes | 5 |
| 82 | F |  | Dabigatran | Unknown | Unknown | Unknown | Unknown | 85 |  |  |  | No |  |
| 74 | M |  | Dabigatran | Unknown | Washout - wound | Wound infection |  | 105 |  |  |  | No |  |
| 59 | M |  | Rivaroxaban | Vascular | Aortic arch replacement | Aortic dissection | High | 124 |  |  | 9 | No |  |
| 75 | M |  | Edoxaban | Vascular | Aortic dissection repair | Aortic dissection | High | 83 |  | 15.9 |  | No |  |
| 63 | M |  | Apixaban | Vascular | Aortic dissection repair | Aortic dissection | High | 128 | 46.1 |  |  | No |  |
| 70 | M |  | Apixaban | Vascular | CVC insertion | Central venous catheter insertion | Minimal | 545 |  |  |  | Yes | 1 |
| 38 | M |  | Rivaroxaban | Vascular | CVC insertion | Central venous catheter insertion | Minimal | 119 |  |  |  | No |  |
| 78 | F |  | Edoxaban | Vascular | CVC insertion | Central venous catheter insertion | Minimal | 509 | 5.5 | 6.3 |  | No |  |
| 78 | F |  | Apixaban | Vascular | CVC insertion | Central venous catheter insertion | Minimal | 170 |  |  |  | Yes | 1 |
| 79 | M | Cholangiocarcinoma | Apixaban | Vascular | CVC removal | Tunneled central venous catheter removal | Minimal | 458 |  |  |  | Yes | 68 |
| 70 | M |  | Apixaban | Vascular | Embolization of splenic artery pseudoaneurysm | Splenic artery pseudoaneurysm | High | 50 |  |  |  | No |  |
| 53 | M |  | Rivaroxaban | Vascular | Femoral pseudoaneurysm - exploration | Infected and bleeding left groin pseudoaneurysm | High | 59 | 0.3 |  |  | Yes | 16 |

**Supplementary Table 3. Selected parameters according to whether patients were treated with high or low-dose four factor prothrombin complex concentrate (4F-PCC) defined as above or below the median dose per kg.** Percentages are calculated as % of patients for whom data was non-missing. Values in parentheses are percentages unless indicated otherwise; *values are median (interquartile range). DOAC: direct oral anticoagulant; IU: international units; RBC: red blood cells. Statistical testing of differences by ^†^t-test (d.f.), ^‡^Mann-Whitney U test, ^#^Fisher’s exact test

|  | <29 IU/kg | | ≥29 IU/kg | |  |
| --- | --- | --- | --- | --- | --- |
|  | n=62 | Missing data (n=, %) | n=65 | Missing data (n=, %) | p |
| Time from last dose of DOAC to reversal (hours)* | 24.0  (20.5 to 28.5) | 17 (27.4) | 23.7  (13.3 to 30.9) | 50 (77.0) | .613^†^ (30.0) |
| Time to reversal to procedure (hours)* | 1.9 (0.7 to 4.3) | 42 (67.7) | 1.5 (0.6 to 4.3) | 40 (61.5) | .900^‡^ |
| Reduced dose DOAC (n, %) | 17 (27.4) | 0 | 15 (23.8) | 2 (3.1) | .686^#^ |
| Bleeding risk of procedure (n, %) |  | 0 |  | 2 (3.1) |  |
| *High (n, %)* | 25 (40.3) |  | 42 |  | **.005**^#^ |
| *Low-moderate (n, %)* | 20 (32.3) |  | 15 |  |  |
| *Minimal (n, %)* | 17 (27.4) |  | 6 |  |  |
| Pre-procedure hemoglobin (g/L)* | 118 (102 to 141) | 2 (3.2) | 116 (97 to 140) | 0 | .339^†^ (119.3) |
| Change in hemoglobin before and after (g/L)* | -10 (-16 to -2) | 16 (25.8) | -8 (-20 to -2) | 10 (1.5) | .983^#^ |
| Red cell transfusion post-procedure | 9 (14.5) | 0 | 10 (15.9) | 2 (3.1) | 1.000^#^ |
| *RBC units** | 2 (1 to 2) | 0 | 2 (1 to 4) | 0 | 1.000^‡^ |
| 90-day mortality post-reversal | 21 (33.8) | 0 | 14 (21.5) | 0 | .167^#^ |
| 90-day bleeding-related mortality post-reversal | 1 (1.6) | 0 | 0 | 0 | .496^#^ |
| 30-day thrombosis post-reversal | 3 (4.8) | 0 | 2 (3.1) | 0 | .680^#^ |

**Supplementary Table 4. Selected parameters according to whether patients were treated with high or low-dose four factor prothrombin complex concentrate (4F-PCC) defined as above or below the median absolute dose.** Percentages are calculated as % of patients for whom data was non-missing. Values in parentheses are percentages unless indicated otherwise; *values are median (interquartile range). DOAC: direct oral anticoagulant; IQR; IU: international units; RBC: red blood cells. Statistical testing of differences by ^†^t-test (d.f.), ^‡^Mann-Whitney U test, ^#^Fisher’s exact test

|  | <2010 IU | | ≥2010 IU | |  |
| --- | --- | --- | --- | --- | --- |
|  | n=84 | Missing data  (n=, %) | n=84 | Missing data  (n=, %) | p |
| Time from last dose of DOAC to reversal (hours)* | 26.9 (13.1 to 33.5) | 69 (82.1) | 23.5 (15.2 to 29.0) | 64 (76.2) | .398^†^ (26.8) |
| Time to reversal to procedure (hours)* | 1.7 (0.5 to 4.3) | 56 (66.7) | 1.5 (0.8 to 4.2) | 57 (67.9) | .860^‡^ |
| Reduced dose DOAC (n, %) | 29 (35.4) | 2 (2.4) | 17 (20.2) | 0 | .056^#^ |
| Bleeding risk of procedure (n, %) |  | 2 (2.4) |  | 0 |  |
| *High (n, %)* | 33 (40.2) |  | 52 (61.9) |  | **.012**^#^ |
| *Low-moderate (n, %)* | 32 (39.0) |  | 21 (25.0) |  |  |
| *Minimal (n, %)* | 19 (23.1) |  | 11 (13.1) |  |  |
| Pre-procedure hemoglobin (g/L)* | 120 (102 to 139) | 4 (4.8) | 119 (97 to 144) | 0 | .807 (160.8) |
| Change in hemoglobin before and after (g/L)* | -9 (-16 to -1) | 22 (26.2) | -10 (-20 to -3) | 16 (19.0) | .254^‡^ |
| Red cell transfusion post-procedure | 14 (17.1) | 2 (2.4) | 8 (9.5) | 0 |  |
| *RBC units** | 2 (1 to 3) | 0 | 2 (1 to 5) | 0 | .770^‡^ |
| 90-day mortality post-reversal | 26 (31.2) | 0 | 19 (22.6) | 0 | .296^#^ |
| 90-day bleeding-related mortality post-reversal | 0 | 0 | 1 (1.2) | 0 | 1.000^#^ |
| 30-day thrombosis post-reversal | 4 (4.9) | 0 | 2 (2.4) | 0 | .682^#^ |
